# A long noncoding RNA with enhancer-like function in pig zygotic genome activation

**DOI:** 10.1093/jmcb/mjae061

**Published:** 2025-01-02

**Authors:** Renyue Wei, Yanbin Yue, Yinhuan Wu, Chenyuan Zhang, Jun-Xue Jin, Zhonghua Liu, Jiaqiang Wang

**Affiliations:** Key Laboratory of Animal Cellular and Genetics Engineering of Heilongjiang Province, College of Life Science, Northeast Agricultural University, Harbin 150030, China; College of Animal Science and Technology, Northeast Agricultural University, Harbin 150030, China; Key Laboratory of Animal Cellular and Genetics Engineering of Heilongjiang Province, College of Life Science, Northeast Agricultural University, Harbin 150030, China; Key Laboratory of Animal Cellular and Genetics Engineering of Heilongjiang Province, College of Life Science, Northeast Agricultural University, Harbin 150030, China; Key Laboratory of Animal Cellular and Genetics Engineering of Heilongjiang Province, College of Life Science, Northeast Agricultural University, Harbin 150030, China; Key Laboratory of Animal Cellular and Genetics Engineering of Heilongjiang Province, College of Life Science, Northeast Agricultural University, Harbin 150030, China; Key Laboratory of Animal Cellular and Genetics Engineering of Heilongjiang Province, College of Life Science, Northeast Agricultural University, Harbin 150030, China; Key Laboratory of Animal Cellular and Genetics Engineering of Heilongjiang Province, College of Life Science, Northeast Agricultural University, Harbin 150030, China

**Keywords:** pre-implantation embryonic development, zygotic genome activation, lncRNA, porcine

## Abstract

The zygotic genome activation (ZGA) is crucial for the development of pre-implantation embryos. Long noncoding RNAs (lncRNAs) play significant roles in many biological processes, but the study on their role in the early embryonic development of pigs is limited. In this study, we identify *lncFKBPL* as an enhancer-type lncRNA essential for pig embryo development. *lncFKBPL* is expressed from the 4-cell stage to the morula stage in pig embryos, and interference with *lncFKBPL* leads to a developmental arrest at the 8-cell stage. Mechanistic investigations uncover that *lncFKBPL* is able to bind to MED8, thereby mediating enhancer activity and regulating *FKBPL* expression. Additionally, FKBPL interacts with the molecular chaperone protein HSP90AA1, stabilizing CDK9 and boosting its protein-level expression. Elevated CDK9 levels enhance Pol II phosphorylation, facilitating ZGA. Our findings illuminate the role of *lncFKBPL* as an enhancer lncRNA in pig ZGA regulation and early embryo development, providing a foundation for further exploration in this area.

## Introduction

Long noncoding RNAs (lncRNAs) are transcripts longer than 200 nucleotides, lacking apparent protein-coding capability (Hansen et al., 2011). lncRNAs have a wide range of functions, and their mechanisms are highly complex, participating in nearly all known biological processes. lncRNAs undergo various processing steps, including capping, polyadenylation, and intron splicing (Rinn et al., 2007). Recent studies have suggested the existence of circular lncRNAs, further adding to their intricate nature (Burd et al., 2010; Hansen et al., 2011). Substantial evidence indicates that lncRNAs play crucial roles in gene expression regulation by directly recruiting epigenetic complexes or influencing the transcription process (Lyon, 1961; Burd et al., 2010). Certain lncRNAs are implicated in diverse biological processes such as epigenetic regulation, pluripotency maintenance, and transcriptional control (Huarte et al., 2010; Khorkova et al., 2015). Moreover, aberrant expression of lncRNAs is associated with numerous diseases, serving as potential biomarkers for various cancers. During embryonic development, lncRNAs contribute to organ differentiation (Grote and Herrmann, 2015) and also influence the aging process (Xing et al., 2017). Although extensive research on lncRNAs has been conducted in mice and humans, studies in other species remain relatively limited. In recent years, numerous studies have reported the identification of lncRNAs in various pig tissues (Li et al., 2016; Liu et al., 2017; Yang et al., 2017; Zhao et al., 2018). Some lncRNAs linked to pig growth performance and reproductive development have been identified (Gao et al., 2017; Zou et al., 2018; Chen et al., 2019). For instance, Hu et al. (2020) identified ovarian lncRNAs in Large White sows during the follicular and luteal phases of the estrous cycle, revealing their association with prolificacy, significantly affecting sow reproductive capability. Liu et al. (2018) investigated the expression profile of lncRNAs in the follicular phase on Day 0, Day 2, and Day 4 in the ovaries of Duroc pigs and identified that the lncRNA ENSSSCT00000034907 may play a significant role in follicular development. However, despite these advancements, there is still a dearth of in-depth research on the functional role of lncRNAs in the early embryonic development of pigs (He et al., 2024).

Mammalian pre-implantation embryos undergo a series of complex changes, including zygotic genome activation (ZGA) (Minami et al., 2007; Schulz and Harrison, 2019), embryo compaction, and the first cell fate differentiation (Mihajlović and Bruce, 2017; Zhang et al., 2018; Yao et al., 2019). These processes necessitate precise regulation to convert terminally differentiated gametes into totipotent cells (Rivera and Ross, 2013; Xu and Xie, 2018). ZGA, as one of the most crucial events initiating new life after fertilization, has been extensively researched. Factors governing the ZGA process encompass the transcriptionally required complex (Poueymirou and Schultz, 1989), members of the heat shock protein family such as HSP70.1 (Christians et al., 1997), the eIF-4C transcription initiation factor (Sonehara et al., 2008), Nr5A2 (Lai et al., 2023; Li et al., 2024), and the OBOX family (Ji et al., 2023). While early embryo development in pigs generally mirrors that in mice, there exist species-specific distinctions. For instance, OCT4 is silent in the trophectoderm (TE) of mice but expressed in the inner cell mass (Ovitt and Schöler, 1998); conversely, in pigs, OCT4 exhibits significant expression in early TE and is crucial for the rapid proliferation of porcine TE (Bou et al., 2022). Moreover, ZGA occurs at 4-cell and 2-cell stages in pigs and mice, respectively (Cao et al., 2014). Mice typically form blastocysts at 3.5 days post-fertilization, while pigs require 6–7 days post-fertilization to reach the blastocyst stage (Bou et al., 2017). The regulatory mechanisms governing ZGA during the early embryonic development of pigs remain relatively understudied.

Endogenous retroviruses (ERVs) are retroviral sequence elements internalized in the genomes of higher jawed vertebrates through evolution. Human ERVs make up ∼8% of the human genome (Lander et al., 2001), while mouse ERVs account for ∼10% of the mouse genome (Waterston et al., 2002). ERVs play specific roles during the growth and development of organisms. The long terminal repeats (LTRs) flanking ERVs often display promoter or enhancer activity (Lu et al., 2014; Engreitz et al., 2016). Furthermore, ERVs can act as mediators, driving the evolution of host genes that counter viral infections or mitigate disease mechanisms. Additionally, owing to their endogenous nature, ERVs introduce new genetic information into the host genome, including unique protein-coding genes and *cis-*regulatory elements (Johnson, 2019). In both mice and humans, >2/3 of lncRNAs are associated with ERVs, playing pivotal roles in numerous cellular processes and diseases (Wang et al., 2016). For instance, the lncRNA *BC005512* is linked to DNA damage repair (Wu et al., 2012), while the morphogenesis of the mammalian placenta involves ERV-related *syncytin-1* (Lavialle et al., 2013).

Moreover, studies have shown that transcripts of MuERV-L are specifically expressed in 2-cell mouse embryos, serving as a marker for 2-cell-like mouse embryonic stem (ES) cells (Macfarlan et al., 2012). Similarly, human ERV with tRNA^His(H)^ serves as a marker for naïve-like human ES cells, participating in the regulating of their pluripotency network (Lu et al., 2014; Ohnuki et al., 2014). This suggests a critical regulatory role for ERVs in early mammalian embryo development. In both mice and humans, ERVs play various pivotal roles during ZGA. For instance, ERVs contribute hundreds of thousands of new regulatory elements to human transcription, with many crucial ZGA-specific genes being regulated by ERV's LTR elements. ERV-related lncRNA *lincGET* can regulate gene transcription and RNA splicing during ZGA, ensuring the normal progression of ZGA (Wang et al., 2016). It forms a complex with CARM1, thereby participating in regulating the first cell fate decision (Wang et al., 2018). However, there remains a dearth of research on ERV-related lncRNAs in the early embryonic development of pigs.

## Results

### *lncFKBPL* is a nucleus-localized lncRNA highly expressed during the pig cleavage stage

**Figure 1:**
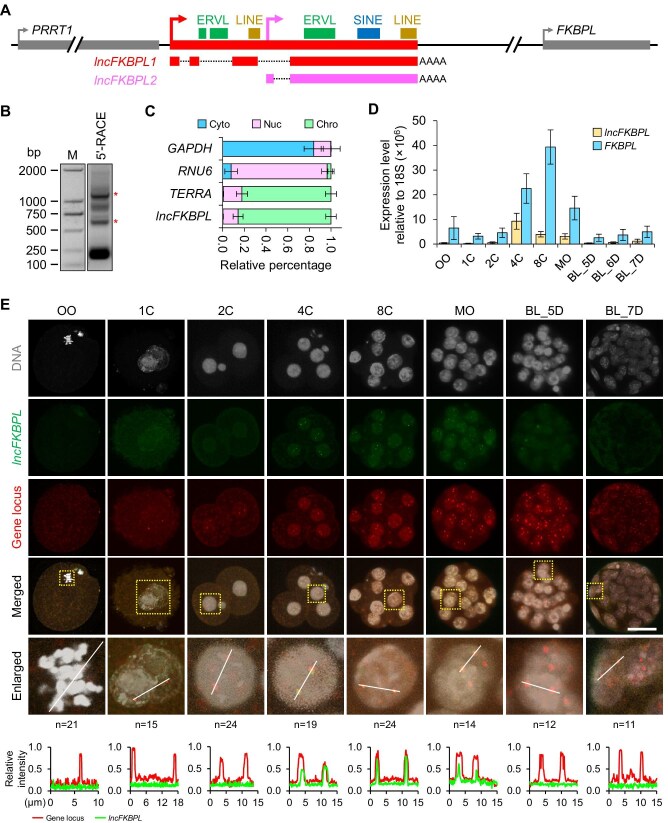
*lncFKBPL* is an ERV-associated nuclear lncRNA highly expressed in pig 4-cell embryos. (**A**) Gene locus of *lncFKBPL. lncFKBPL* is between *PRRT1* and *FKBPL* and has two transcript isoforms, *lncFKBPL1* and *lncFKBPL2*. There are some ERVL, SINE, and LINE fragments on *lncFKBPL* gene locus. AATAAA is the polyadenylated signal site. (**B**) 5′-RACE results for *lncFKBPL*. Primers are shown in Supplementary Figure S1A. The asterisk (*) indicates the bands corresponding to the correct band of 5′-RACE for *lncFKBPL*. Approximately 200 pig 4-cell embryos were used for each experiment and three experimental replicates were performed. M, DNA ladder. (**C**) Subcellular localization analysis of *lncFKBPL* by RNA fractionation and qPCR. The error bars represent standard error of the mean (SEM). *GAPDH, RNU6*, and *TERRA* act as cytoplasm (Cyto), nucleoplasm (Nuc), and chromosome (Chro) control, respectively. Approximately 500 pig 4-cell embryos were used for each experiment and three experimental replicates were performed. (**D**) Expression pattern of *lncFKBPL* and *FKBPL* at different stages of pig pre-implantation embryos analyzed by qPCR. The error bars represent SEM. About 50 embryos of each stage were used and three experimental replicates were performed. OO, MII oocytes; 1C, 1-cell embryos; 2C, 2-cell embryos; 4C, 4-cell embryos; 8C, 8-cell embryos; MO, morula; BL_5D, blastocysts at embryonic day 5; BL_6D, blastocysts at embryonic day 6; BL_7D, blastocysts at embryonic day 7. (**E**) RNA-FISH combined with DNA-FISH in pig oocytes and early embryos to detect *lncFKBPL* transcripts and *lncFKBPL* gene locus. Three experimental replicates were performed. Top, representative images. Scale bar, 50 μm. One nucleus (marked by the square) is magnified in the ‘enlarged’ lane. Bottom, line scans of the relative intensity of fluorescence signals indicated by the white lines.

To identify lncRNAs associated with ERVs during early pig embryo development, we utilized reverse transcription with primers containing polyT tails, followed by polymerase chain reaction (PCR) amplification using semi-random primers targeting ERVs and adapter primers (Figure 1A; Supplementary Figure S1A and Table S1). Subsequently, we ligated the PCR products to a T vector and performed transformation, selecting 20 individual clones for Sanger sequencing. Upon alignment with the UCSC website, we identified 14 transcripts, with the highest proportion being a transcript located between the *PRRT1* and *FKBPL* genes (Supplementary Table S2). We then conducted rapid amplification of cDNA ends (RACE) analysis on this transcript and discovered two isoforms, designated as *lncFKBPL1* and *lncFKBPL2* (Figure 1B). To verify the expression of *lncFKBPL*, we examined the 3′-end of the *PRRT1* gene, the upstream gene of *lncFKBPL*, as its transcription might overlap with *lncFKBPL* (Supplementary Figure S1A). The 3′-RACE results indicated that the 3′-end of the *PRRT1* gene did not overlap with the *lncFKBPL* gene (Supplementary Figure S1B). Repeat sequence analysis revealed the presence of multiple transposon sequences, including transposon elements from ERV with tRNA^Leu(L)^ (ERVL), long interspersed nuclear elements (LINE), and short interspersed nuclear elements (SINE), on the locus of *lncFKBPL*, indicating it as an ERV-associated transcript.

To further investigate the functional role of *lncFKBPL* as a lncRNA, we conducted coding potential prediction for *lncFKBPL1* and *lncFKBPL2*, which indicated a low likelihood of translation (Supplementary Figure S1C). Additionally, subcellular localization analysis revealed that *lncFKBPL* is primarily localized to the chromosome (Figure 1C). Considering that translation occurs in the cytoplasm, these findings suggest that *lncFKBPL* is a nucleus-localized lncRNA. Fluorescence real-time quantitative PCR (qPCR) results revealed that *lncFKBPL* is expressed from the 4-cell stage to the morula stage of pig embryos, with peak expression observed at the 4-cell stage (Figure 1D). Besides, the level of *lncFKBPL1* is higher than *lncFKBPL2* at 4-cell and 8-cell stages (Supplementary Figure S1D). To validate the results of subcellular localization analysis and expression pattern analysis, we conducted RNA fluorescence *in situ* hybridization (RNA-FISH) experiments, which demonstrated punctate signals of *lncFKBPL* localized within the cell nucleus, with at most two signal points per nucleus (Figure 1E). Besides, we analyzed the expression levels of *lncFKBPL* and *FKBPL* using the published RNA sequencing (RNA-seq) data (PRJNA783716) (Zhang et al., 2024). The results showed that *lncFKBPL* was expressed in 4-cell and 8-cell embryos, while *FKBPL* was expressed in oocytes and pre-implantation embryos in a gradually increasing pattern (Supplementary Figure S1E and F). This slightly differs from the qPCR results (Figure 1D), possibly due to noticeable variations between different sequencing replicates with low sample sizes (Supplementary Figure S1E and F). Based on these results, we hypothesize that *lncFKBPL* likely remains tethered to its own gene locus after transcription. To confirm this hypothesis, we performed DNA-FISH and RNA-FISH co-staining, revealing co-localization of *lncFKBPL* RNA signals with DNA signals, indicating that *lncFKBPL* does not detach from the gene locus after transcription (Figure 1E). This suggests that *lncFKBPL* may exert its function through a *cis-*regulatory mechanism.

### *lncFKBPL* depletion leads to developmental arrest at 4- to 8-cell stages

**Figure 2:**
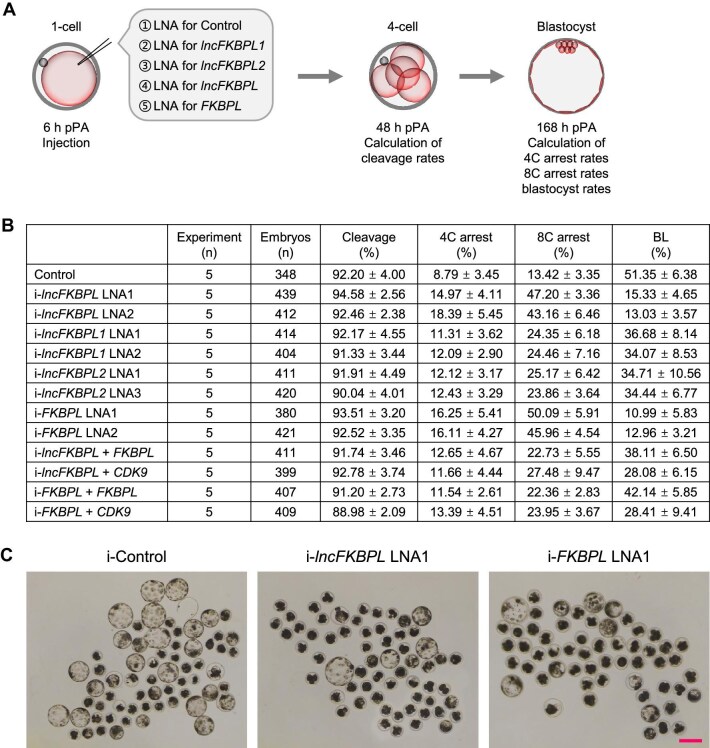
*lncFKBPL* or *FKBPL* depletion results in developmental arrest at 4- to 8-cell stages. (**A**) The experimental scheme to analyze the effects of *lncFKBPL, lncFKBPL1, lncFKBPL2*, or *FKBPL* depletion on embryonic development. (**B**) Statistics for embryonic development after microinjection of control LNA (i-Control), LNA targeting *lncFKBPL* (i-*lncFKBPL*), LNA targeting *lncFKBPL1* (i-*lncFKBPL1*), LNA targeting *lncFKBPL2* (i-*lncFKBPL2*), LNA targeting *FKBPL* (i-*FKBPL*), LNA targeting *lncFKBPL* and *FKBPL* mRNA (i-*lncFKBPL* + *FKBPL*), LNA targeting *lncFKBPL* and *CDK9* mRNA (i-*lncFKBPL* + *CDK9*), LNA targeting *FKBPL* and *FKBPL* mRNA (i-*FKBPL* + *FKBPL*), or LNA targeting *FKBPL* and *CDK9* mRNA (i-*FKBPL* + *CDK9*). Student's *t*-test was used for statistical analysis, and the *P*-values are shown in Supplementary Figure S2B. (**C**) Microcopy of embryos at blastocyst stage. Scale bar, 100 μm. Five experimental replicates were performed for each group.

To probe the involvement of *lncFKBPL* in early pig embryo development, we injected interfering fragments targeting *lncFKBPL* at the 1-cell stage. While the cleavage rate was unaffected by *lncFKBPL* interference (Figure 2; Supplementary Figure S2), early embryo development was impacted, evidenced by a significant decrease in blastocyst formation rate (Figure 2; Supplementary Figure S2B). Embryos were primarily arrested at the 8-cell stage, with some degree of arrest also observed at the 4-cell stage (Figure 2; Supplementary Figure S2). These findings indicate the crucial regulatory role of *lncFKBPL* in early pig embryo development, as *lncFKBPL* depletion resulted in developmental arrest at 4- to 8-cell stages.

### *lncFKBPL* facilitates upregulation of downstream gene *FKBPL* expression

**Figure 3:**
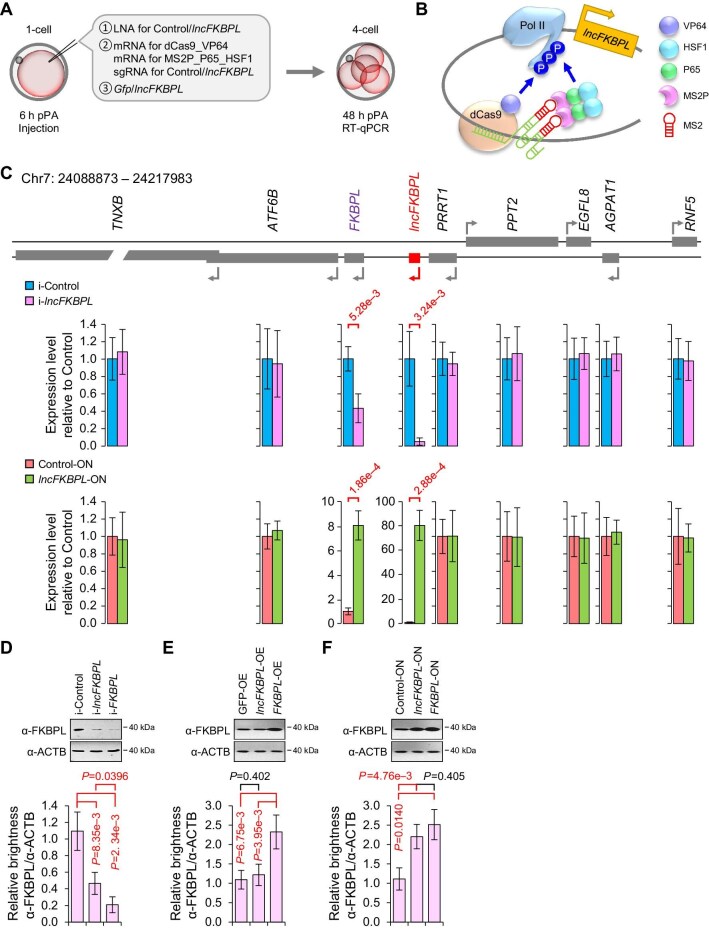
*lncFKBPL* activates *FKBPL in cis*. (**A**) The experimental scheme to analyze the effects of interference, overexpression, or endogenous activation of *lncFKBPL* on neighbor genes. (**B**) The illustration of endogenous activation of *lncFKBPL* via the CRISPR-ON system. (**C**) Top, illustration of genes around *lncFKBPL* on pig chromosome 7 (Chr7: 24088873–24217983). Gene locus of *lncFKBPL* is shown in red. Middle, changes of gene expression levels upon *lncFKBPL* depletion analyzed by qPCR. Bottom, changes of gene expression levels upon endogenous activation of *lncFKBPL* through the CRISPR-ON system analyzed by qPCR. The error bars represent SEM. Student's *t*-test was used for statistical analysis. (**D**–**F**) Western blotting and quantification of FKBPL protein levels upon depletion (**D**), overexpression (**E**), or endogenous activation through the CRISPR-ON system (**F**) of *lncFKBPL* or *FKBPL*. ACTB works as an internal reference. Three experimental replicates were performed. The error bars represent SEM. Student's *t*-test was used for statistical analysis.

Considering that *lncFKBPL* does not detach from its gene locus after transcription (Figure 1E), we sought to unravel the mechanism by which *lncFKBPL* regulates early pig embryo development. Initially, we investigated the impact of *lncFKBPL* interference on the expression levels of surrounding genes (Figure 3A and B). Results indicated a significant decrease in the expression of the downstream gene *FKBPL* following *lncFKBPL* interference, while the expression of other nearby genes remained unaffected (Figure 3C and D; Supplementary Table S1). This suggests a positive regulatory role of *lncFKBPL* in *FKBPL* expression. To further ascertain whether *lncFKBPL* regulation of *FKBPL* is *cis-*regulatory, we separately injected *lncFKBPL* RNA (overexpression *in trans*) and employed the CRISPR-ON system to induce endogenous activation of *lncFKBPL* (overexpression *in cis*, Figure 3B) in early pig embryos. Results revealed that overexpression *in trans* failed to activate *FKBPL* expression (Figure 3E; Supplementary Figure S3), whereas overexpression *in cis* activated both endogenous *lncFKBPL* and *FKBPL* expression (Figure 3C and F). Additionally, neither overexpression *in trans* nor *in cis* affected the expression of surrounding genes. These findings demonstrate that *lncFKBPL* activates *FKBPL* expression through a *cis-*regulatory mechanism.

### *lncFKBPL* mediates enhancer activity to activate *FKBPL* expression

In 2010, De Santa et al. (2010) identified 3216 lncRNAs, with 2236 (69%) found within enhancer regions. It was shown that enhancer-derived *lncRNAs* have the ability to activate the expression of surrounding target genes *in cis*. As *lncFKBPL* activates *FKBPL* expression through *cis-*regulation, we hypothesized that *lncFKBPL* might function as an enhancer-type lncRNA.

**Figure 4:**
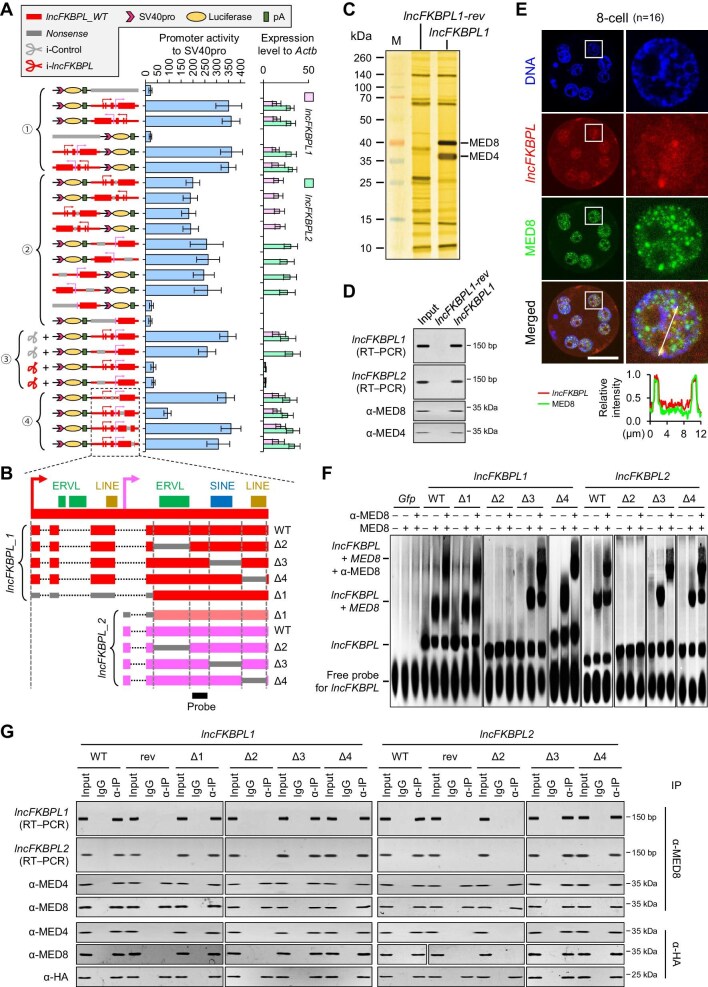
*lncFKBPL* has an enhancer-like function. (**A**) The results of the dual-luciferase reporter system. The y-axis shows the construction of luciferase reporter plasmids and LNAs. ① *lncFKBPL* is forwardly or reversely inserted upstream or downstream of luciferase reporter gene. ② Promoter regions of *lncFKBPL1* and/or *lncFKBPL2* are replaced with nonsense sequences to disrupt their transcription. ③ Control LNAs or LNAs targeting *lncFKBPL* are overexpressed to deplete *lncFKBPL1* and *lncFKBPL2*. ④ Different regions of *lncFKBPL* are replaced with nonsense sequences to generate different *lncFKBPL* deletion mutants. Three experimental replicates were performed. The error bars represent SEM. Student's *t*-test was used for the statistical analysis, and the *P*-values are shown in Supplementary Figure S4A. (**B**) Illustration of *lncFKBPL* deletion mutants. Four different regions were deleted individually, resulting in seven types of *lncFKBPL* transcripts: *lncFKBPL-Δ1, lncFKBPL_1-Δ2, lncFKBPL_1-Δ3, lncFKBPL_1-Δ4, lncFKBPL_2-Δ2, lncFKBPL_2-Δ3*, and *lncFKBPL_2-Δ4*. (**C**) Silver staining of SDS-PAGE gel following the RNA pull-down assay shows the proteins bound to *lncFKBPL_1* (right lane) and *reverse lncFKBPL_1* (*lncFKBPL_1-rev*, middle lane). Only one pull-down assay for mass spectrometry analysis was performed with 3271 pig 8-cell embryos. Two specific bands in the right lane were analyzed by mass spectrometry and confirmed to be MED4 and MED8. M, protein ladder. (**D**) Western blotting following the RNA pull-down assay. Three experimental replicates were performed. (**E**) Top, lncFKBPL and MED8 co-stained by immunofluorescence combined with RNA-FISH in pig 8-cell embryos. One nucleus (marked by square) is magnified in the right lane. Bottom, line scans of the relative intensity of fluorescence signals indicated by the white dotted line. (**F**) Results of RNA EMSA. Three biological replicates were performed. (**G**) Co-IP followed by reverse transcription–PCR and western blotting using PEFs expressing HA-tagged MS2P and MS2-labelled *lncFKBPL*. Three biological replicates were performed.

To confirm our hypothesis, we initially examined whether *lncFKBPL* exhibits enhancer activity using a dual-luciferase reporter system. The results revealed that regardless of its insertion upstream or downstream of the luciferase reporter gene, and irrespective of forward or reverse insertion, *lncFKBPL* significantly enhanced luciferase expression (Figure 4A, ①; Supplementary Figure S4A), indicating its enhancer activity. This led to a new question: whether the gene sequence of *lncFKBPL* itself possesses enhancer activity, or its transcription product mediates enhancer activity. To address this, we replaced the promoter region of *lncFKBPL1* and/or *lncFKBPL2* with a nonsense sequence from the neomycin resistance gene. The results demonstrated that the level of luciferase expression significantly decreased when only *lncFKBPL1* or *lncFKBPL2* was expressed, while the luciferase expression was lost when neither *lncFKBPL1* nor *lncFKBPL2* was expressed (Figure 4A, ②; Supplementary Figure S4A). Moreover, solely expressing *lncFKBPL2* resulted in a slightly higher luciferase expression level compared to solely expressing *lncFKBPL1*, potentially due to the higher transcriptional activity of *lncFKBPL2* in the reporter plasmid (Figure 4A, ②; Supplementary Figure S4A). Additionally, co-transfection of locked nucleic acid (LNA) targeting *lncFKBPL* disrupted its enhancer activity (Figure 4A, ③; Supplementary Figure S4A). These results indicate that the enhancer activity of *lncFKBPL* is mediated by its transcription product, confirming *lncFKBPL* as an enhancer-type lncRNA that activates the expression of target genes by mediating enhancer activity.

Next, we aimed to identify which segment of *lncFKBPL* mediates enhancer activity. To address this question, we mutated *lncFKBPL* based on the repetitive sequences, obtaining four mutant sequences for each isoform (Figure 4B). The luciferase reporter results showed that when the ERVL sequence in the exon of *lncFKBPL* was mutated to a nonsense sequence (Δ2), the luciferase expression level significantly decreased, while the other three mutations did not cause a significant change in luciferase expression levels (Figure 4A, ④, and B; Supplementary Figure S4A). These results indicate that the region containing the ERVL sequence in the exon of *lncFKBPL* is the core region mediating enhancer activity.

To explore the mechanism by which *lncFKBPL* mediates enhancer activity, we conducted pull-down followed by mass spectrometry experiments to analyze the protein interactors of *lncFKBPL*. We found that *lncFKBPL* specifically bound to MED4 and MED8 (Figure 4C; Supplementary Figure S4B and C), which was confirmed by pull-down followed by western blotting (Figure 4D). MED4 and MED8 are components of the Mediator complex (Supplementary Figure S4D), which primarily functions to mediate the interaction between transcription factors binding to enhancers and RNA polymerase binding to promoters (Allen and Taatjes, 2015). The Mediator complex is mainly divided into four main parts: the head, middle, tail, and CDK kinase module (Supplementary Figure S4D; Soutourina, 2018). The results of RNA-FISH combined with immunofluorescence experiments demonstrated that *lncFKBPL* was co-localized with MED4 and MED8 in pig 8-cell embryos (Figure 4E; Supplementary Figure S4E).

We further investigated which region of *lncFKBPL* interacts with MED4/MED8. RNA electrophoretic mobility shift assay (EMSA) results showed that the addition of MED8 caused a band shift in the wild-type *lncFKBPL*, and further band shifts were observed when both MED8 and anti-MED8 antibody were added, confirming the binding of *lncFKBPL* to MED8 (Figure 4F). Additionally, after mutation of the ERVL region in the exon (Δ2), MED8 failed to cause band shifts in *lncFKBPL*, indicating that this mutant cannot interact with MED8 (Figure 4F). These results indicate that the ERVL region in the exon of *lncFKBPL* can bind to MED8. To verify the above results, we overexpressed *lncFKBPL* with MS2 tags and MS2-binding protein with HA tags in porcine embryonic fibroblasts (PEFs) (Pickett and Peabody, 1993) and conducted RNA immunoprecipitation (IP) and co-immunoprecipitation (co-IP) experiments (Figure 4G). In PEFs expressing wild-type *lncFKBPL, lncFKBPL* was precipitated by both anti-MED4 and anti-MED8 antibodies, and both MED4 and MED8 were precipitated by anti-HA antibody, indicating their mutual binding. However, in cells expressing the *lncFKBPL* mutant without the ERVL region (Δ2), both anti-MED4 and anti-MED8 antibodies failed to precipitate the *lncFKBPL* mutant, and anti-HA antibody failed to precipitate MED4 or MED8. These results demonstrate that *lncFKBPL* can indeed form a complex with MED4 and MED8, with the ERVL region being necessary. Additionally, MED4 was still precipitated by anti-MED8 antibody even in cells expressing the *lncFKBPL* mutant Δ2, indicating that *lncFKBPL* is not necessary for the interaction between MED4 and MED8 (Figure 4G).

### *FKBPL* forms a complex with *HSP90AA1* and stabilizes *CDK9*

**Figure 5:**
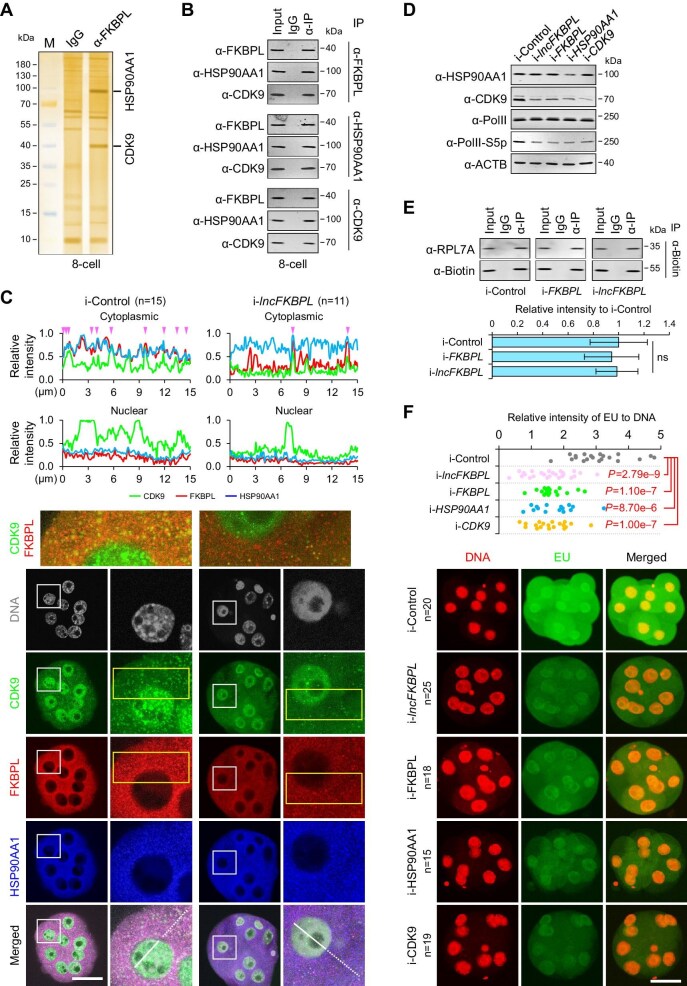
FKBPL forms a complex with HSP90AA1 to stabilize CDK9 and then promote ZGA. (**A**) Silver staining of SDS-PAGE gel following the co-IP assay shows the proteins bound to FKBPL (right lane) and IgG (middle lane). Only one pull-down assay for mass spectrometry analysis was performed with 2472 pig 8-cell embryos. Two specific bands in the right lane were analyzed by mass spectrometry and confirmed to be HSPAA1 and CDK9. (**B**) Western blotting following the co-IP assay. Three biological replicates were performed. (**C**) Bottom, CDK9, FKBPL, and HSPAA1 co-stained by immunofluorescence in pig 8-cell embryos upon *lncFKBPL* depletion. One nucleus marked by the square is magnified in the right lane. The co-localization of CDK9 and FKBPL (marked by the yellow rectangle) is highlighted by merging images with green and red fluorescence. Top, line scans of the relative intensity of fluorescence signals indicated by the dotted lines (cytoplasmic) or lines (nuclear). (**D**) Western blotting results of pig 8-cell embryos upon *lncFKBPL, FKBPL, HSP90AA1*, or *CDK9* depletion. Three biological replicates were performed. The quantification results are shown in Supplementary Figure S5C. (**E**) Western blotting and quantification following the RNA pull-down assay. Three biological replicates were performed. The error bars represent SEM. Student's *t*-test was used for statistical analysis. ns, *P* > 0.05. (**F**) Representative images of EU staining and relative intensity of EU signals in pig 8-cell embryos upon *lncFKBPL, FKBPL, HSP90AA1*, or *CDK9* depletion. Student's *t*-test was used for statistical analysis, and the *P*-values are shown.

To elucidate the function of FKBPL, we injected mRNA encoding HA-tagged FKBPL into parthenogenetic embryos at the 1-cell stage and collected embryos at the 8-cell stage. Subsequently, we conducted IP followed by mass spectrometry experiments using anti-HA antibody to explore proteins interacting with FKBPL. The results revealed that FKBPL can bind to HSP90AA1 and CDK9 (Figure 5A; Supplementary Figure S5A and B). IP followed by western blotting confirmed the formation of a complex between FKBPL, HSP90AA1, and CDK9 (Figure 5B). Furthermore, immunofluorescence results showed that FKBPL and HSP90AA1 exhibited similar localization patterns in pig 8-cell embryos, i.e. primarily in the cytoplasm and concentrated around the nucleus, with significant co-localization (Figure 5C). Additionally, CDK9 was mainly localized in the nucleus but also present in a small amount in the cytoplasm, and cytoplasmic CDK9 was co-localized with FKBPL and HSP90AA1 (Figure 5C). These results indicate that FKBPL can form a complex with HSP90AA1 and CDK9.

HSP90AA1 is a molecular chaperone, and previous studies have suggested that the interaction between FKBPL and HSP90AA1 promotes the stability of associated proteins (McKeen et al., 2010). Thus, we aimed to investigate whether the FKBPL–HSP90AA1 complex enhances the stability of CDK9. Our results revealed a significant reduction in CDK9 protein levels upon *FKBPL* ablation, while the RNA levels of *CDK9* remained unaffected (Figure 5D; Supplementary Figure S5C–E). To further evaluate the translation efficiency of *CDK9* following *FKBPL* depletion, we injected biotin-labelled *CDK9* and siRNAs targeting *FKBPL* into pig 1-cell embryos. Ribosome binding efficiency on *CDK9* was then assessed using co-IP with an anti-biotin antibody. Interestingly, there was no significant alteration in the translation efficiency of *CDK9* upon *FKBPL* depletion (Figure 5E). Furthermore, after *lncFKBPL* interference, the localization and protein level of HSP90AA1 remained unaffected, while the protein level of CDK9 significantly decreased (Figure 5C and D; Supplementary Figure S5C). Notably, the translation efficiency of *CDK9* was not affected upon *lncFKBPL* ablation (Figure 5E). These findings collectively suggest that the FKBPL–HSP90AA1 complex upregulates CDK9 at the protein level by promoting protein stability.

### CDK9 promotes activation of porcine zygotic genes through Pol II phosphorylation

CDK9 is an important subunit of the CDK kinase module of the Mediator complex, capable of phosphorylating RNA polymerase II (Pol II) to activate downstream gene expression. Therefore, we hypothesized that in early pig embryos, after FKBPL and HSP90 increase CDK9 expression at the protein level, CDK9 would promote Pol II phosphorylation, thus facilitating porcine ZGA. Western blotting results showed that interference with *lncFKBPL, FKBPL, HSP90AA1*, or *CDK9* led to decreased levels of Pol II phosphorylation (Figure 5D; Supplementary Table S1). Consistently, immunofluoresence results showed that the Pol II phosphorylation level decreased upon depletion of *lncFKBPL, FKBPL, HSP90AA1*, or *CDK9* (Supplementary Figure S6). These results indicated that the *lncFKBPL*–FKBPL–HSP90AA1–CDK9 axis indeed promotes Pol II phosphorylation.

**Figure 6:**
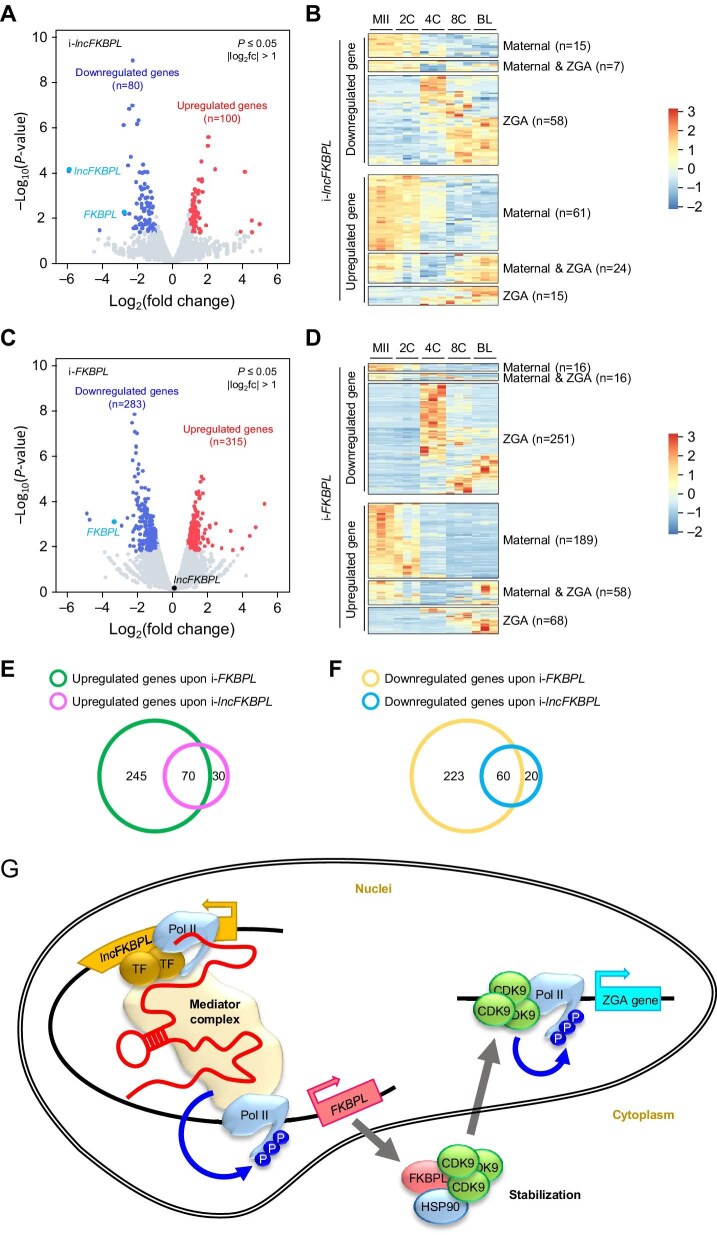
*lncFKBPL* promotes pig ZGA. (**A**–**D**) Volcano plots and heatmaps depicting differentially expressed genes upon lncFKBPL interference (i-lncFKBPL, **A** and **B**) or FKBPL interference (i-FKBPL, **C** and **D**). (**E** and **F**) Venn diagrams depicting the intersection of upregulated genes (**E**) or downregulated genes (**F**) between i-lncFKBPL and i-FKBPL. (**G**) Model of *lncFKBPL* function in pig ZGA. In pig 4- to 8-cell embryos, *lncFKBPL* interacts with MED4 and MED8, components of the Mediator complex, and has an enhancer-like function to activate *FKBPL in cis*. FKBPL forms a complex with HSP90AA1 to stabilize CDK9, which promotes phosphorylation of Pol II and then promotes ZGA.

Subsequently, we used 5-ethynyl uridine (EU) staining to assess the activation level of porcine zygotic genes in 8-cell embryos and found that interference with *lncFKBPL, FKBPL, HSP90AA1*, or *CDK9* resulted in decreased EU staining levels (Figure 5F). Furthermore, RNA-seq on samples with *lncFKBPL* or *FKBPL* interference showed that *lncFKBPL* depletion resulted in 100 upregulated and 80 downregulated genes, while *FKBPL* depletion resulted in 315 upregulated and 283 downregulated genes (Figure 6A and B). Further analysis, combined with published RNA-seq data (PRJNA783716) (Zhang et al., 2024), revealed that the downregulated genes were mainly ZGA genes, whereas the upregulated ones were primarily maternal genes, upon either *lncFKBPL* or *FKBPL* depletion (Figure 6C and D). Notably, the differentially regulated genes upon *lncFKBPL* depletion and *FKBPL* depletion largely overlapped, i.e. 70 out of 100 upregulated genes (Figure 6E) and 60 out of 80 downregulated genes (Figure 6F), suggesting that *lncFKBPL* and *FKBPL* share a similar regulatory network. These results collectively suggest the involvement of the *lncFKBPL*–FKBPL–HSP90AA1–CDK9 axis in the regulation of porcine ZGA (Figure 6G).

## Discussion

In this study, we report a chromatin-localized lncRNA, termed *lncFKBPL*, which is expressed during porcine ZGA. We found that interference with *lncFKBPL* leads to developmental arrest of pig embryos at 4- to 8-cell stages, indicating the crucial importance of *lncFKBPL* in early pig embryo development. Mechanistic studies revealed that *lncFKBPL* activates downstream gene *FKBPL* expression *in cis* by mediating enhancer activity, with its exon ERVL sequence interacting with MED8 to facilitate its enhancer function. We discovered that FKBPL forms a complex with HSP90AA1, thereby stabilizing CDK9 and promoting CDK9 protein level. CDK9, in turn, promotes Pol II phosphorylation, thereby facilitating porcine ZGA. This study provides new insights into porcine ZGA.

Enhancer lncRNAs are a common regulatory model for lncRNA function. Studies have shown that the deletion of most lncRNAs leads to decreased expression levels of nearby protein-coding genes, indicating that the majority of lncRNAs possess enhancer-like functions (De Santa et al., 2010; Orom and Shiekhattar, 2013). Here, we identified *lncFKBPL* as an enhancer model regulated by *cis-*acting mechanisms to control the expression of the downstream gene *FKBPL*. This is the first discovery of an enhancer model lncRNA in early pig embryo development.

In addition, we found that, during early pig embryo development, FKBPL interacts with HSP90AA1 and CDK9, stabilizing CDK9, which in turn phosphorylates Pol II to activate the expression of downstream zygotic genes. Current research on FKBPL mainly focuses on its role as a tumor growth and angiogenesis inhibitor in breast and ovarian cancers. FKBPL is a member of the immunophilin protein family, exhibiting anti-angiogenic properties and the ability to inhibit endothelial cell migration and blood vessel formation. Studies have shown that FKBPL acts as a cytoplasmic regulator in estrogen receptor signaling by interacting with HSP90 (McKeen et al., 2010). In light of our findings, FKBPL likely acts as a molecular chaperone, synergistically regulating the stability of substrate proteins with HSP90. Sengun et al. (2021) collected DNA samples from multiple males and conducted mutation analysis using Sanger sequencing, revealing that mutations in the *FKBPL* gene may lead to male infertility. Our study elucidates the mechanism by which *lncFKBPL–FKBPL* promotes porcine ZGA through HSP90AA1 and CDK9, expanding the understanding of FKBPL’s functions. Whether *lncFKBPL* is expressed in other tissues or organs of pigs and whether the mechanism of its enhancer activity in activating *FKBPL* also exists in other tissues or organs remain to be further explored. Additionally, whether this mechanism is conserved in other mammals also warrants further investigation.

Recently, it has been reported that phase separation at super-enhancers of coactivators, including the components of the Mediator complex, plays essential roles in gene expression control (Boija et al., 2018; Chong et al., 2018; Sabari et al., 2018). Besides, repeat-containing RNAs have the capability to accumulate into abnormal foci in the nucleus through phase separation (Jain and Vale, 2017). Thus, we speculated that the ERVL repeat-containing RNAs, including *lncFKBPL*, might regulate phase separation of MED4 and MED8, which will be investigated in our future work.

The three-dimensional genome organization plays a crucial role in determining how genes are regulated, where chromosomes are compartmentalized into topologically associated domains (TADs) (Rao et al., 2014; Pombo and Dillon, 2015). The gene(s) can interact with regulatory elements located in the same TAD, while interactions across different TADs are prevented by TAD boundaries that are often bound by specific proteins including CTCF and cohesin complexes (Hong and Kim, 2017). This may explain why *lncFKBPL* specifically enhances the expression of *FKBPL* in the genome of early pig embryos (Figure 3C), while *lncFKBPL* promotes luciferase expression both upstream and downstream when tested in a plasmid system (Figure 4A). Future research utilizing chromatin conformation capture assays, such as Hi-C, and chromatin immunoprecipitation with anti-CTCF or anti-cohesin antibodies would be valuable for investigating this possibility.

## Materials and methods

### Primer and probe design

Primers were designed using Premier5, and both primers and FISH probes were synthesized by the Beijing Genomics Institute (Beijing, China).

### Construction of plasmid vectors

To create Alexa Fluor™ 488-labelled RNA probes for *lncFKBPL* RNA-FISH, the specific *lncFKBPL* region (1847–2039) was amplified using the 2× Vazyme Lamp Master Mix (Dye Plus) (Vazyme, P312). For co-IP experiments, the MS2 coat protein (MS2P), MS2-labelled *lncFKBPL*, and HA-labelled MS2P were cloned into the PB533A vector (SBI, PB533A-2) digested with *Eco*RI or *Sal*I, respectively.

### Antibodies

The following antibodies were used for western blotting, EMSA, and co-IP assays: anti-MED4 (Thermo, MA5-42613), anti-MED8 (Thermo, PA5-118854), anti-FKBPL (Thermo, PA5-99231), anti-HSP90AA1 (Thermo, MA5-45157), anti-CDK9 (Abcam, ab239364), anti-POLII-S5p (Abcam, ab5131), anti-POLII (Abcam, ab5131), anti-RPL7A (Abcam, ab70753), anti-Biotin (Abcam, ab201341), anti-ACTB (Santacruz, sc47778), and anti-HA (Abcam, ab1424).

### Embryo culture and collection

Ovaries were collected from a local slaughterhouse and preserved in physiological saline at 37°C during transportation. The ovaries were washed with 37°C physiological saline containing penicillin and streptomycin. Cumulus–oocyte complexes (COCs) were aspirated from follicles with a diameter of 3–6 mm using a 10-ml syringe with an 18-gauge needle and washed three times with HEPES buffer. COCs with uniform cytoplasm and at least three layers of tightly packed cumulus cells were selected for *in vitro* maturation. Fifty COCs were placed in 500 μl of Tissue Culture Medium 199 (Invitrogen, 31100-027) containing 0.14% polyvinyl alcohol, 10 ng/ml epidermal growth factor, 0.57 mM cysteine, 0.5 IU/ml pregnant mare serum gonadotropin (PROSPEC, hor272), and 0.5 IU/ml human chorionic gonadotropin (PROSPEC, hor250). COCs were matured at 39°C for 42–44 h. After maturation, COCs were transferred to an Eppendorf tube containing 0.1% hyaluronidase, and surrounding cumulus cells were removed using a manual pipette. Oocytes with a polar body were selected for parthenogenetic activation (PA). PA embryos were cultured in PZM3 medium, which included 500 μl PZM-3 containing 0.15% bovine serum albumin, 200 μl BME amino acid solution (Merck, B6766), and 100 μl MEM non-essential amino acid solution (Merck, M7145).

Embryos were collected at the following time points post-PA (pPA): 1-cell stage (pPA 12 h), 2-cell stage (pPA 24 h), early 4-cell stage (pPA 40 h), mid 4-cell stage (pPA 48 h), late 4-cell stage (pPA 56 h), 8-cell stage (pPA 72 h), blastocyst stage (pPA 108 h), early blastocyst stage (pPA 120 h), mid blastocyst stage (pPA 144 h), and late blastocyst stage (pPA 168 h).

### Culture cells and transfection

PEFs and PK15 cells were cultured in a fibronectin-coated dish in a fibroblast culture medium consisting of 15% fetal bovine serum (BI, 04-002-1A), 1% non-essential amino acids (Invitrogen, 11140050), 1% glutamax (Gibco, 35050061), 1% penicillin–streptomycin–glutamine (Gibco, 10378016), and Dulbecco's modified Eagle's medium (Gibco, 11965092) at 37°C in a 5% CO_2_ incubator. The culture medium was changed every day. PEFs and PK15 cells were passaged every 2–3 days and then digested to single cells by 0.05% trypsin/EDTA (Gibco, 25300062). For cell transfection, PEFs were passaged and seeded at a density of 10000–15000 cells/cm^2^. After 2 days (60%–70% confluence), plasmid DNA with or without LNA targeting *lncFKBPL* was transferred into cells using Lipofectamine™ 3000 transfection reagent (Gibco, L3000015) according to the manufacturer's instructions. PEFs were collected for further analysis after 36 and 72 h, respectively.

### RNA extraction, reverse transcription, and qPCR

RNA was extracted using the RNeasy Mini Kit (QIAGEN, 74104), and the RNase-Free DNase Set (QIAGEN, 79254) was used to prevent DNA contamination. Reverse transcription was carried out using RevertAid™ (Thermo, M1631) with the specified parameters, and the resulting cDNA was stored at −20°C until needed. The analysis of gene expression through qPCR was performed using TB Green® Premix Ex Taq™ (TaKaRa, RR420A). All these kits were used following the manufacturers’ guidelines.

### CRISPR-ON

For the preparation of the mRNA of dCas9 fused with transcription activating domain VP64 (dCas9_VP64) and the mRNA of MS2P fused with transcription activating domains P65 and HSF1 (MS2P_P65_HSF1), DNA fragment containing T7 promoter and dCas9_VP64 and DNA fragment containing T7 promoter and MS2P_P65_HSF1 were amplified from the plasmid dCAS9-VP64_GFP (Addgene, 61422) and the plasmid MS2-P65-HSF1_GFP (Addgene, 61423) using LongAmp^TM^ Taq DNA Polymerase (NEB, M0534L), respectively. Then, *in vitro* transcription was performed using the HiScribe™ T7 Quick High Yield RNA Synthesis Kit (NEB, E2050). For the preparation of sgRNAs targeting promoters of *lncFKBPL1* and *lncFKBPL2*, spacer sequences were designed on the website of CRISPR RGEN Tools (http://www.rgenome.net/cas-designer/), and DNA fragments containing T7 promoter and MS2-labelled sgRNAs were generated from the plasmid sgRNA(MS2) (Addgene, 61424). Then, *in vitro* transcription was performed with the HiScribe™ T7 High Yield RNA Synthesis Kit (NEB, E2040). Primers are shown in Supplementary Table S1.

More details for routine assay methods are listed in Supplementary Materials and methods.

## Supplementary Material

mjae061_Supplemental_File
