# Supplementary material for: A long noncoding RNA with enhancer-like function in pig zygotic genome activation
Source: J Mol Cell Biol. 2025 Jan 2;17(1):mjae061. doi: 10.1093/jmcb/mjae061 (PMC12210014; doi:10.1093/jmcb/mjae061)
Supplement: mjae061_Supplemental_File [file mjae061_supplemental_file.pdf]

Supplementary material

A long noncoding RNAs with enhancer-like function in pig zygotic genome activation

Supplementary Materials and methods

RNA fractionation

About 500 pig four-cell embryos were lysed with 100 μL of pre-chilled RLN1 solution (50 mM Tris-HCl pH 8.0, 140 mM NaCl, 1.5 mM $MgCl_2$, 0.5% NP-40, and 2 mM Vanadyl Ribonucleoside Complex) for 5 minutes on ice. After centrifugation at 4 ℃, 300 g for 2 minutes, the supernatant was transferred to new RNase-free tube as the cytoplasmic fraction. The precipitate was lysed with 100 μL of pre-chilled RLN2 solution (50 mM Tris-HCl pH 8.0, 500 mM NaCl, 1.5 mM $MgCl_2$, 0.5% NP-40, and 2 mM Vanadyl Ribonucleoside Complex) for 5 minutes on ice. After centrifugation at 4 ℃, 18000 g for 2 minutes, the supernatant was transferred to new RNase-free tube as the nucleoplasm fraction, and the precipitate was treated as the chromatin fraction. Total RNA extraction was performed using the RNeasy Mini Kit (QIAGEN, 74104) and eluted with the same volume of elution buffer to ensure comparability. Reverse transcription and qPCR were conducted for quantitative analysis, with GAPDH as the cytoplasmic localization control, Xist as the chromatin localization control, and RNU6 as the nucleoplasm localization control.

DNA-FISH combined with RNA-FISH

For the preparation of probes in DNA-FISH, 17 DNA fragments containing T7 promoter (2,100 – 2,700 bp) were amplified from the mouse genome using LongAmpTM Taq DNA Polymerase (NEB, M0534L). Primers were shown in Supplementary Table 1. Then *in vitro* transcription was performed with mixed 17 DNA fragments using the MEGAshortscript™ Kit (Ambion, AM1354) with ATP, CTP, GTP, UTP, and ChromaTide™ Alexa Fluor™ 488-5-UTP (Invitrogen, C11403) solution (4: 4: 4: 1: 4) in which 80% of uracil was labelled by Alexa Fluor™ 488. Then the labelled RNA was fragmented by adding 1 × Ambion RNA fragmentation reagent (Ambion, AM8740) with incubation at 70 ℃ for 2 min. After adding the stop solution, the labelled RNA was purified and used as probes for DNA-FISH.

The zona pellucida of the mouse embryos was removed with incubation in acidic Tyrode's Solution. Then the embryos were incubated in PBSA for 3 min and transferred onto Superfrost/Plus microscope slides and dried as quickly as possible (less than 5 min). Embryos were permeabilized in

1% Triton X-100 in 1× PBS and fixed in ice-cold 100% methanol for 30 min at -20 ℃. Then, slides were transferred into 70% ethanol on ice for 20 min. To perform RNA-FISH, the procedures for dehydration and hybridization were performed as in IF combined with the RNA-FISH section. After three washes for 5 min each in hybridization washing solution at 42 ℃ and four washes for 5 min each in 2× SSC, slides were post-fixed in 3% paraformaldehyde (PFA, Sigma, 158127) in PBS for 10 min at room temperature.

To perform DNA-FISH, the embryos were incubated with RNase Cocktail (Invitrogen, AM2288) in 1× PBS for one hour at 37 ℃. After three washes with 1× PBS, embryos were permeabilized in permeabilization solution II (0.7% Triton X-100 and 0.1M HCl in 1× PBS) for 15 min on ice. Then, slides were transferred into 70% ethanol on ice for 20 min. Dehydration was performed in 80%, 95%, and 100% ethanol (×2), with each incubation lasting for 5 min at room temperature. Slides were then dried for 5 min. Then the slides were denatured in the hybridization washing solution for 30 min at 80 ℃. After dehydration in cold ethanol, the embryos were hybridized in the hybridization solution containing 5 μg of Alexa Fluor™ 546-labelled DNA probes per slide at 37 ℃ overnight (14-15 hours). After three washes for 5 min each in hybridization washing solution (50% Formamide, 2× SSC) at 42 ℃ and four washes for 5 min each in 2× SSC, embryos were mounted with DAPI-Vectashield solution (Vector laboratories, H1200). Fluorescence staining was imaged using a laser-scanning confocal microscope (Leica, TCS SP8). IMARIS software (Bitplane) was then used to calculate the number of speckles for each picture.

Microinjection

To downregulate or overexpress lncRNA and mRNA, nucleotide acids (locked nucleotide acids at 10 μM, mRNA at 150 ng/μL, sgRNA at 60 ng/μL) was injected into the cytoplasm of mature oocytes using the FemtoJet microinjector (Eppendorf; Hamburg, Germany). Under conditions of injection pressure at 150 hPa, compensation pressure at 50 hPa, and injection time of 0.7 seconds, the same amount of DNA was injected into each embryo. The injection volume for each embryo was approximately 10 pL. The transport of RNA in mature pig oocytes was performed 6 hours after parthenogenetic activation (PA). This process was carried out on a heating platform under an inverted microscope (Nikon Corporation; Tokyo, Japan), with microinjection performed in MAN buffer medium.

EU staining

At pPA 56 h, EU (10 mM, final concentration) was added to pig embryos injected with i-NC, i-lncFKBPL, i-FKBPL, i-HSP90AA1, or i-CDK9, respectively. The embryos were then cultured for 16 hours. After removing the zona pellucida with acidic Tyrode's solution, the pig eight-cell stage

embryos were washed twice with washing solution for 5 minutes each. Subsequently, embryos were fixed in 4% PFA in 1× PBS for 30 minutes, followed by permeabilization using a standard permeabilization solution. The binding of E was detected using the Click-iT RNA Alexa Fluor 488 Imaging Kit (Invitrogen, C10329).

Western blot analysis

Each group consisted of a total of 200 pig embryos, which were washed twice with PBSA. They were then lysed in 60 μL lysis buffer (20mM HEPES, 150mM NaCl, 2mM EGTA, 1mM EDTA, 20mM glycerol phosphate, 1% TritonX-100, and 10% glycerol), containing 0.6 μL PMSF (100mM, Beyotime) for 2 hours, followed by boiling at 100 ℃ for 5 minutes. Proteins were separated on a 12% ExpressPlusTM PAGE gel (GenScript), transferred onto a nitrocellulose membrane (Millipore), and then detected using immunoblotting. After blocking the membrane with 5% BSA in TBST for 2 hours at room temperature, it was incubated overnight at 4 ℃ with primary antibodies. Following three washes with TBST, the membrane was incubated with an HRP-conjugated secondary antibody. For Western blot analysis, bands were visualized using SuperSignal™ West Pico PLUS (Thermo, 34577) according to the manufacturer's instructions.

RNA electrophoretic mobility shift assays (REMSA)

RNA oligonucleotides were *in vitro* transcribed using HiScribe™ T7 Quick High Yield RNA Synthesis Kit (NEB, E2050) following the manufacturers' guidelines. RNA oligonucleotides were then biotinylated using the Pierce RNA 3′ End Desthiobiotinylation Kit (Thermo, 20163) according to the manufacturer's instructions. The labelled oligonucleotides were gel purified on 12% denaturing gels before use. The gel shift assay was carried out using the LightShift Chemiluminescent RNA EMSA Kit (Thermo, 20158). Briefly, 5 ng biotinylated wildtype or mutant RNA probe was mixed with 50 μg of 6×His-purified PCBP1 and PCBP2 mix (6×His-PCBP1) (and an-PCBP1 or anti-PCBP2 antibody for super-shifts) and incubated at room temperature for 30 min in a 20 μL binding reaction containing 1× binding buffer, 5% glycerol and 0.1 mg/ml tRNA. The samples were electrophoresed on a 5% native PAGE in 0.5× Tris Borate EDTA (Thermo, B52), transferred to a positively charged BrightStar®-Plus Nylon membrane (Invitrogen, AM10100), and crosslinked in a UV Stratalinker 1800 (Stratagene). To block the membrane, the membrane was incubated in 20 ml Nucleic Acid Detection Blocking Buffer for 15 min with gentle shaking, and then was incubated in 20 ml conjugate/blocking solution (20 ml Nucleic Acid Detection Blocking Buffer containing 66.7 μL Stabilized Streptavidin-Horseradish Peroxidase Conjugate) for 15 min with gentle shaking. After washing four times for 5 min each in 20 ml 1× Wash Buffer with gentle shaking, the membrane was incubated in 30 ml Substrate Equilibration Buffer for 5 min with gentle shaking. Then the membrane

was incubated in 12 ml Substrate Working Solution (6 ml Luminol/Enhancer Solution and 6 ml Stable Peroxide Solution) for 5 min without shaking. Finally, the membrane was exposed to a Bio-Rad GelDoc XR+ Gel imaging system for the acquisition of signals.

Double Luciferase assay

The plasmid TK with pGL3-luciferase vectors associated with SV40 promoter and wild type *lncFKBPL* (lncFKBPL_WT) or *lncFKBPL* mutants (lncFKBPL_Mut) were co-transfected with or without LNA targeting *lncFKBPL* into PEF cells. After culturing for 48 hours, the culture medium was removed and added PBS to wash the cells twice. Then the fluorescence intensity was measured using the dual-luciferase reporter analysis system (Promega, E1910).

Immunofluorescence staining

Fix pig embryos in 4% paraformaldehyde (PFA) at room temperature for 30 minutes. Then, wash the embryos three times for 5 minutes each in a washing solution (0.1% Tween-20, 0.01% Triton X-100, dissolved in 1× PBS). After washing, block the embryos with a blocking solution (1% BSA in 1× PBS) at room temperature for 1 hour, followed by overnight incubation at 4℃ with a primary antibody diluted in the blocking solution. After washing three times for 5 minutes each in a washing solution, incubate the embryos with a secondary antibody labeled with the Alexa series fluorescence, diluted in the washing solution, at room temperature for 1 hour. After washing three times in the washing solution, stain the cell nuclei with DAPI (10 mg/mL, dissolved in 1× PBS) for 7 minutes. Following three additional washes, directly image the embryos in microdrops in the culture dish using a laser scanning inverted confocal microscope (LSM 780).

Immunoprecipitation (IP)

Two million pig fibroblast cells or approximately 3000 pig eight-cell embryos were lysed using 500 μL of immunoprecipitation lysis buffer (0.025M Tris, 0.15M NaCl, 0.001M EDTA, 1% NP40, 5% glycerol). After incubating on ice for 5 minutes, the lysate was transferred to a new centrifuge tube and centrifuged at 13000g for 10 minutes. The supernatant was transferred to a new centrifuge tube for protein concentration determination and subsequent experiments. Pierce Protein A/G Magnetic Beads vial was vortexed to obtain a homogeneous suspension. 25 μL of beads were added to the centrifuge tube. The tube was placed on a magnetic rack for 1 minute to collect the beads. The supernatant was removed, and 500 μL of 1×modified coupling buffer (0.1 mL 20× crosslinking buffer, 0.1 mL immunoprecipitation lysis buffer, 19.8 mL deionized water) was added to the centrifuge tube. Gentle mixing was done, and the tube was incubated at room temperature on a rotator for 1 minute. The beads were collected using a magnetic rack, and the supernatant was removed. This step was

repeated once. The antibody was diluted 1:20 in 20× coupling buffer and immunoprecipitation lysis buffer to achieve a final antibody concentration of 5 μg per 100 μL. 100 μL of prepared antibody solution was added to the beads, gently mixed, and incubated at room temperature on a rotator for 15 minutes. During incubation, the beads were gently vortexed every 5 minutes to keep them in suspension. The beads were collected using a magnetic rack, and the supernatant was removed. 100 μL of 1× modified coupling buffer was added, mixed, and the beads were collected using a magnetic rack, and the supernatant was removed. 300 μL of 1× modified coupling buffer was added, and the beads were gently vortexed or inverted for mixing. The beads were collected using a magnetic rack, and the supernatant was removed. This step was repeated once. The sample lysate was diluted to 500 μL with immunoprecipitation lysis buffer. The diluted lysate (500 μL) was added to the centrifuge tube containing antibody-coupled beads and incubated overnight at 4°C. 500 μL of immunoprecipitation lysis/wash buffer was added to the centrifuge tube, gently mixed, and the beads were collected, and the supernatant was discarded. This process was repeated once. 500 μL of deionized water was added to the tube, gently mixed, and the beads were collected using a magnetic rack, and the supernatant was discarded. 100 μL of elution buffer was added to the centrifuge tube. After mixing, the tube was incubated on a shaker at room temperature for 5 minutes. The beads were separated by magnetic force, and the supernatant containing the target antigen was retained. After elution, 10 μL of neutralizing solution was added to every 100 μL of elution buffer to neutralize the low pH. To obtain more antigen, elution can be repeated once.

Pull-down assay

First, perform in vitro transcription and add poly A. This part of the procedure refers to the mMESSAGEmMACHINE® T7 ULTRA Kit (Ambion, AM1345). Linearize 10 μg of plasmid DNA at the single enzyme cutting site located at the 3' end of the insert fragment (incubate at 37 ℃ for 5 hours). Add Proteinase K to a final concentration of 0.5 μg/μl, and incubate at 37°C for 45 minutes to remove all RNA enzymes. Extract DNA using an equal volume mixture of phenol/chloroform (1:1) at room temperature, centrifuge at 12000g for 10 minutes, and transfer the upper aqueous phase to a RNase-free centrifuge tube. Add 1/10 volume of 3M sodium acetate and 2.5 times the volume of anhydrous ethanol, cool at -80 ℃ for 30 minutes. Centrifuge at 12000g, 4 ℃ for 10 minutes to precipitate DNA, wash with 1 mL of 70%-80% ethanol, and air dry at room temperature for 15 minutes. Dissolve DNA in 10 μl of RNase-free water. Use 1 μg of template, mix 10 μL of 2×NTP/CAP, 2 μL of 10× Reaction Buffer, and 2 μL of T7 Enzyme Mix, and bring to a total volume of 20 μL with water, then incubate at 37°C for 3 hours. Add 1 μL of TURBO DNAse and incubate at 37°C for 15 minutes to complete in vitro transcription. Then, add 20 μL of 5×E-PAP Buffer, 10 μL of 25mM $MnCl_2$, 10 μL of ATP Solution, 36 μL of Nuclease-free Water, and 4 μL of E-PAP enzyme, mix well,

and incubate at 37 ℃ for 45 minutes.

After that, RNA recovery is performed by adding 100 μL of RNase-free water and adjusting the total volume to 200 μL, followed by the addition of 200 μL of chloroform and thorough mixing. Centrifuge at 4 ℃, 15,000 rpm for 5 minutes, then transfer the supernatant (aqueous phase) to a new centrifuge tube. Add an equal volume of chloroform again, mix thoroughly, and centrifuge at 4 ℃, 15,000 rpm for 5 minutes. Transfer the supernatant (aqueous phase) to a new centrifuge tube. Add an equal volume of chloroform, shake well for 5 to 10 seconds. Centrifuge at 4 ℃, 15,000 rpm for 5 minutes, then transfer the supernatant (aqueous phase) to a new centrifuge tube. Add 1/10 volume of 3 M Sodium Acetate, 4 μL of Dr. GenTLE Precipitation Carrier, and 2.5 times the volume of ethanol to the supernatant, mix thoroughly. After incubating at -80 ℃ for 1 hour, centrifuge at 4 ℃, 15,000 rpm for 30 minutes, gently pour off the supernatant, and retain the precipitate. Wash the precipitate with 80% ethanol. After air drying, dissolve the recovered RNA in 8 μL of RNase-free water.

Next, perform 3′ end biotinylation using the Pierce RNA 3′ End Desthiobiotinylation Kit (Thermo, 20163). In a PCR tube, add 8 μL of the in vitro transcribed RNA, heat at 85 ℃ for 3-5 minutes, then immediately place on ice. Add 3 μL of 10×RNA Ligase Reaction Buffer, 1 μL of RNase Inhibitor, 1 μL of Biotinylated Cytidine Bisphosphate, 2 μL of T4 RNA Ligase, and 15 μL of 30% PEG, mix well, and incubate at 16°C for 2 hours for biotinylation of the 3′ end of RNA. Then, add 70 μL of RNase-free water, adjust the total volume to 100 μL, and add 200 μL of chloroform, mix well. Centrifuge at 4 ℃, 15,000 rpm for 5 minutes, and transfer the supernatant (aqueous phase) to a new tube. Avoid touching the interphase. Add 10 μL of 5M NaCl, 1 μL of glycogen, and 300 μL of anhydrous ethanol. After incubating at -80 ℃ for 1 hour, centrifuge at 4 ℃, 15,000 rpm for 30 minutes, gently pour off the supernatant, and retain the precipitate. Wash the precipitate with 300 μL of 70% ethanol. After air drying, dissolve the obtained Bio-RNA in 10 μL of RNase-free water.

The RNA-protein pull-down experiment was conducted using the Pierce Magnetic RNA-Protein Pull-Down Kit (Thermo, 20164). First, 50 μL of Magnetic Beads were aspirated into a 1.5 mL centrifuge tube and placed on a Magnetic Stand for 30 seconds to remove the supernatant. Then, 50 μL of 20 mM Tris (pH 7.5) was added, mixed well, placed on the Magnetic Stand for another 30 seconds, and the supernatant was removed. This step was repeated once. Subsequently, 50 μL of 1 ×RNA Capture Buffer was added and mixed. 50 pmol of Bio-RNA was treated at 90 ℃ for 2 minutes, cooled on ice for 2 minutes, and then slowly equilibrated to room temperature over 20 minutes using RNA structure buffer (containing 10 mM Tris-HCl pH 7.0, 100 mM KCl, and 10 mM $MgCl_2$) to allow the RNA to form its native secondary structure. The processed Bio-RNA was then added to the centrifuge tube containing Magnetic Beads, mixed well, and incubated at room temperature on a shaker for 15-30 minutes. Pierce IP Lysis Buffer (Thermo, 87787) was used to lyse pig eight-cell embryos and obtain the lysate. Place the centrifuge tube containing the processed Bio-RNA on the

Magnetic Stand for 30 seconds, then remove the supernatant. Add 60 μL of 20 mM Tris (pH 7.5), mix well, place it on the Magnetic Stand for another 30 seconds, and remove the supernatant. Repeat this step once. Next, add 100 μL of 1×Protein-RNA Binding Buffer, mix well, place it on the Magnetic Stand for 30 seconds, and remove the supernatant. Add 100 μL of Master Mix (10 μL 10×Protein-RNA Binding Buffer, 30 μL 50% glycerol, 30 μL Lysate, Nuclease-free water), mix well, and incubate on a shaker at 4 ℃ for 60 minutes. Place the centrifuge tube on the Magnetic Stand for 30 seconds, then remove the supernatant. Add 100 μL of 1×Wash Buffer, mix well, place it on the Magnetic Stand for 30 seconds, and remove the supernatant. Repeat this step twice. Add 50 μL of Elution Buffer and incubate on a shaker at 37 ℃ for 15-30 minutes. Place the centrifuge tube on the Magnetic Stand for 30 seconds and collect the supernatant. Incubate at 95-100 ℃ for 5-10 minutes. Part of the obtained RBPs will be used for Western blot, and another part will be used for Mass spectrometry.

After running the gel with RBPs using SDS-PAGE, rinse the gel in deionized water. Then, place the gel in a container filled with 50 mL of fixative (containing 20 mL of EtOH, 5 mL of acetic acid, and 25 mL of deionized water) and fix on a shaker for 20 minutes. Discard the fixative and add 50 mL of 30% ethanol to the shaker for 10 minutes. Remove the 30% ethanol and add 50 mL of Sensitizing solution (containing 15 mL of EtOH, 5 mL of Sensitizer, and 30 mL of deionized water) and incubate on the shaker for 10 minutes. Discard the Sensitizing solution, rinse with 50 mL of 30% ethanol for 10 minutes on the shaker, and then place the gel in a container with 50 mL of deionized water for 10 minutes on the shaker. Next, place the gel in a container with 50 mL of Staining solution (containing 0.5 mL of Stainer and 49.5 mL of deionized water) and incubate on the shaker for 15 minutes. Discard the staining solution, rinse with 50 mL of deionized water for 30 seconds on the shaker. Then, place the gel in a container with 50 mL of Developing solution (containing 5 mL of Developer, 1 drop of Developer enhancer, and 45 mL of deionized water) and incubate on the shaker for 6 minutes until bands appear. Once the staining is sufficient, immediately add 10 mL of stop solution to the container and shake for 10 minutes. Discard the solution, add 50 mL of deionized water, and treat for 10 minutes. Use a clean blade to cut out the desired bands and place them in a 1.5 mL centrifuge tube. Add 50 μL of Destainer A and 50 μL of Destainer B, mix well, and incubate at room temperature for 15 minutes. Remove the liquid, add 200 μL of deionized water, and incubate at room temperature for 10 minutes. Repeat the previous step twice. Finally, use the obtained samples for Mass spectrometry.

RNA-seq data analysis

To trim the raw data, the *trim_galore* (v0.6.10) software was used with default parameters. Next, *STAR* (v2.7.11a) was used to align the clean data to the genome with the parameters: --*outSAMstrandField intronMotif --twopassMode Basic* and the mapped reads were counted for each

gene using *featureCounts* (v2.0.6) with default parameters according to the *gtf* file which is downloaded from ENSMBL and added the *lncFKBPL* annotations according to the RACE result. The differential expressed gene analysis was performed using *DESeq2* (R4.0.5) between the i-Control and i-*lncFKBPL* groups as well as the i-Control and i-*FKBPL* groups, with a selection criterion of p-value < 0.05 and $|\log_2 FoldChange| > 1$. R package *ggplot2* (R3.4.2) was used to generate the volcano plots with these differentially expressed genes. The expression of these differentially expressed genes in MII to blastocyst stage was displayed with heatmap drawn by *pheatmap* (R1.0.12).

Statistical analysis

Statistical analyses [mean ± standard error of the mean (SEM)] for differential gene expression, differential fluorescence intensity, and differential abundance on gels were performed in Excel. Levels of significance were calculated with Student's t-tests. Isoform abundance on SDS-PAGE gels or agarose gels was measured in Fiji/ImageJ. The co-localization analysis of lncFKBPL signals with FKBPL, MED4, MED8, CDK9, HSP90AA1 gene locus in RNA-FISH combined with immunofluorescence assays, and RNA-FISH combined with DNA-FISH assays were calculated by the two (green and red) or three (green, red, and blue) separated channels signals per nucleus using Pearson's correlation coefficient with Coloc 2 plugins in Fiji/ImageJ. Line scans of the relative fluorescence intensity of signals were drawn by separated channels signals with Plot Profile plugins in Fiji/ImageJ. For fluorescence intensity analysis of EU staining (Figure 5F), the whole embryo regions for EU signal and DNA signal were separately cut out, discolored, inverted, and lined in one picture using Photoshop, then analyzed by Image J, and finally the ratio of factor signal intensity to DNA signal intensity was used.

Supplementary Reference

Zhang, D., Wu, D., Zhang, S., et al. (2024). Transcription factor AP-2 gamma affects porcine early embryo development by regulating epigenetic modification. Reprod Biomed Online *49*, 103772.

Supplementary Figures

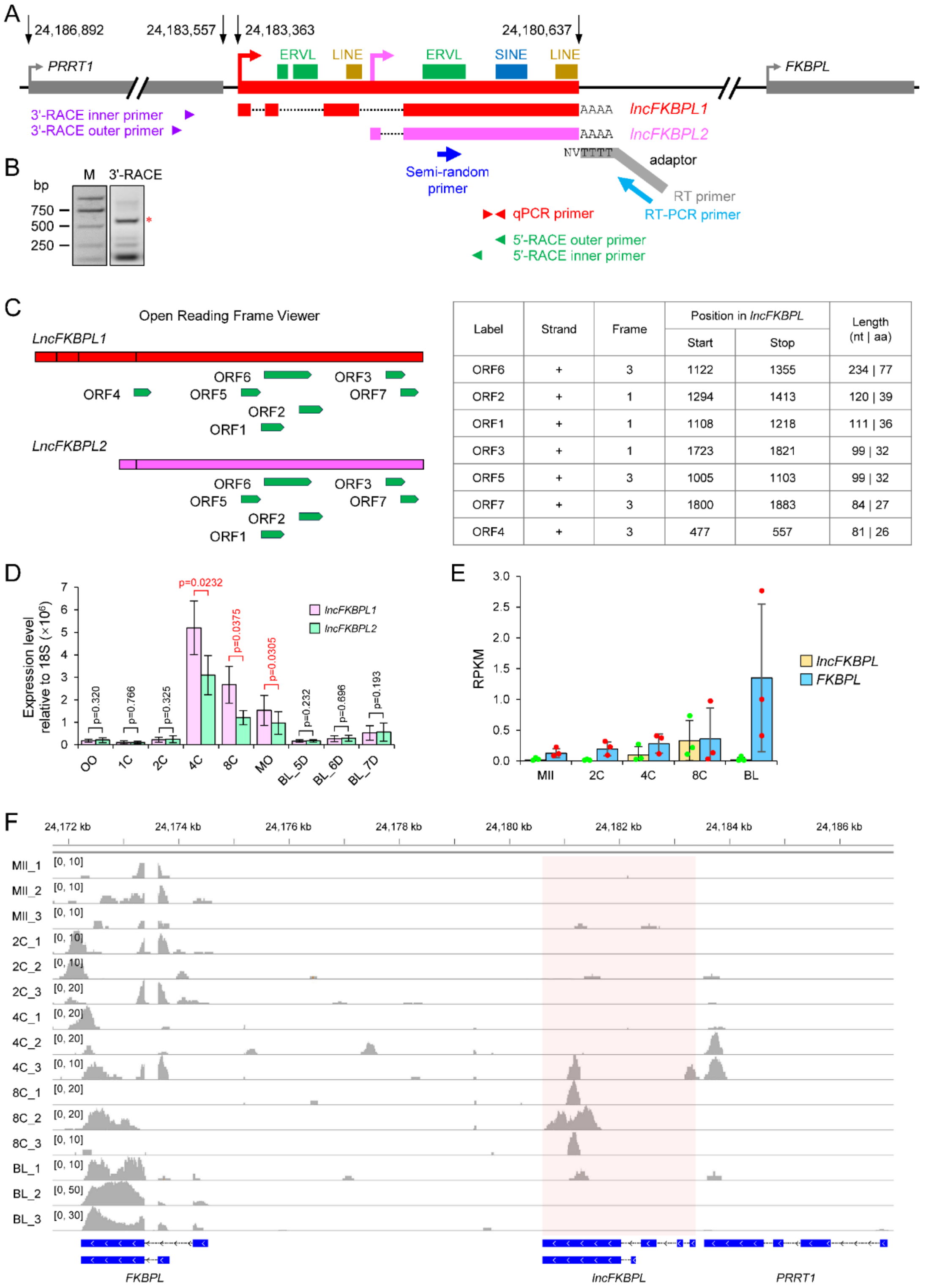

Supplementary Figure S1 *lncFKBPL* is an ERV-associated long non-coding RNA.

A. Gene locus of *lncFKBPL*. *lncFKBPL* is between *PRRT1* and *FKBPL* and has two transcript isoforms with poly(A) tail, *LncFKBPL1* and *lncFKBPL2*. The genome locations of *PRRT1* and *lncFKBPL* are shown. There are some ERVL, SINE, and LINE fragments on *lncFKBPL* gene locus. ERVL, transposon elements from endogenous retrovirus with $tRNA^{Leu(L)}$. SINE, short interspersed nuclear elements. LINE, long interspersed nuclear elements. Adaptor anchored oligo-dT primer (5′-

adaptor-T30VN-3′, where “N” is A, T, C, or G and “V” is A, C, or G) is used for reverse transcription (RT). Primers for RT-PCR, qPCR, and 5'-RACE are shown.

B. 3′-RACE results for *PRRT1*. Primers are shown in panel A. * indicates the band corresponding to the correct band of 3′-RACE for *PRRT1*. About 200 pig four-cell embryos were used for each experiment and three experimental replicates were performed. M, DNA ladder.

C. View of open reading frames of *lncFKBPL* predicted by NCBI.

D. Expression pattern of *lncFKBPL1* and *lncFKBPL2* at different stages of pig preimplantation embryos analyzed by qPCR. The error bars represent SEM. About 50 embryos of each stage were used and three experimental replicates were performed. OO, MII oocytes. 1C, one-cell embryos. 2C, two-cell embryos. 4C, four-cell embryos. 8C, eight-cell embryos. MO, morula. BL_5D, blastocysts at embryonic day 5. BL_6D, blastocysts at embryonic day 6. BL_7D, blastocysts at embryonic day 7. Student’s *t*-test was used for the statistical analysis.

E, F. Expression patterns of *FKBPL* and *lncFKBPL* showed through reads per kilobase per million mapped reads (RPKM) (**E**) and integrative genomics viewer (IGV) screenshots (**F**) at different stages of pig preimplantation embryos analyzed by the published RNA-seq data (PRJNA783716) (Zhang et al., 2024). The error bars in panel E represent SEM. IGV, MII, MII oocyte. 2C, two-cell embryos. 4C, four-cell embryos. 8C, eight-cell embryos. BL, blastocysts.

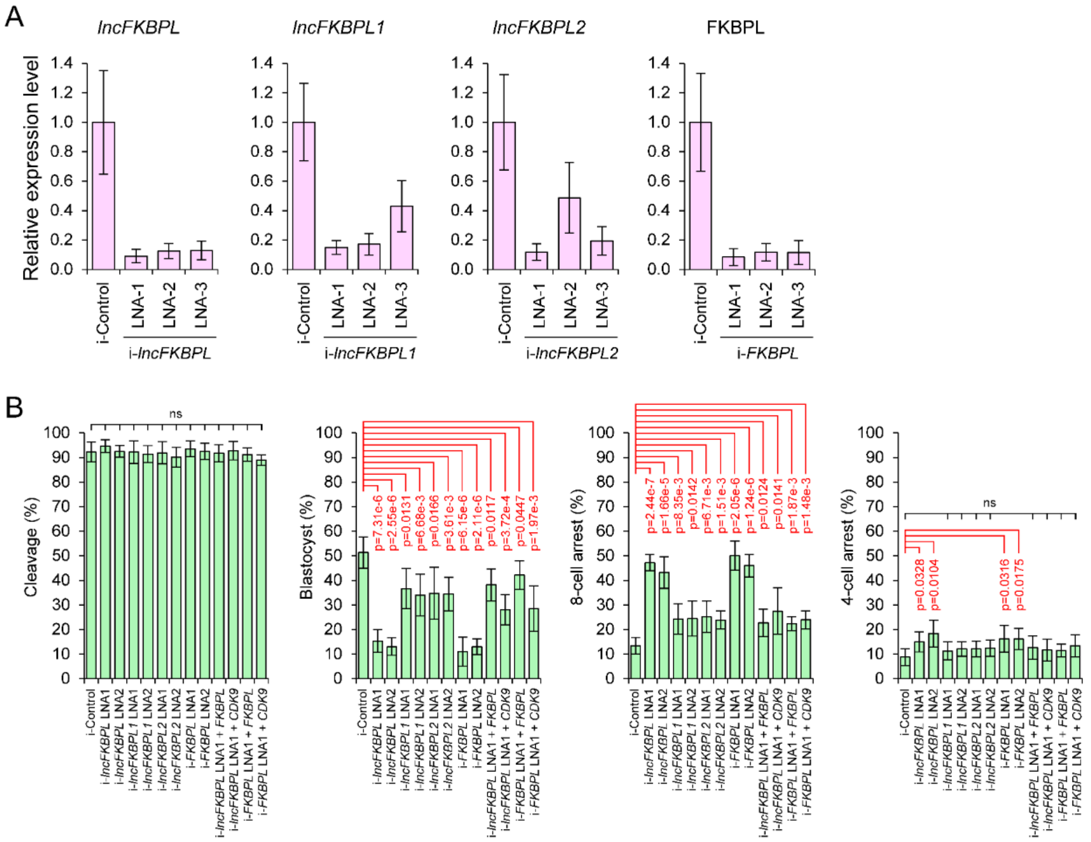

Supplementary Figure S2 Embryonic development upon depletion of *lncFKBPL* or *FKBPL*

A. RNA interference efficiency of LNA on *lncFKBPL*, *lncFKBPL1*, *lncFKBPL2*, and *FKBPL*. Three biological replicates were performed. The error bars represent SEM.

B. Statistic of Cleavage rate, blastocyst rate, eight-cell stage arrest rate, and four-cell stage arrest rate. i-Control, microinjection of control LNA. i-*lncFKBPL*, microinjection of LNA targeting *lncFKBPL*. i-*lncFKBPL1*, microinjection of LNA targeting *lncFKBPL1*. i-*lncFKBPL2*, microinjection of LNA targeting *lncFKBPL2*. i-*FKBPL*, microinjection of LNA targeting *FKBPL*. i-*lncFKBPL* + *FKBPL*, microinjection of LNA targeting *lncFKBPL* and *FKBPL* mRNA. i-*lncFKBPL* + *CDK9*, microinjection of LNA targeting *lncFKBPL* and *CDK9* mRNA. i-*FKBPL* + *FKBPL*, microinjection of LNA targeting *FKBPL* and *FKBPL* mRNA. i-*FKBPL* + *CDK9*, microinjection of LNA targeting *FKBPL* and *CDK9* mRNA. The error bars represent SEM. Student's *t*-tests were used for statistical analysis. ns, $p > 0.05$.

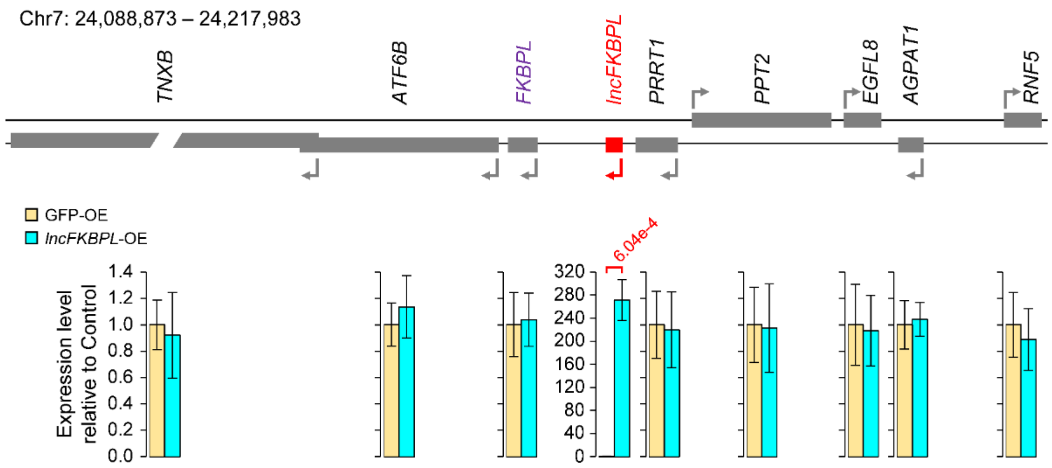

Supplementary Figure S3 *lncFKBPL* overexpression cannot activate *FKBPL*.

Top, illustration of genes around *lncFKBPL* on pig chromosome 7 (Chr7: 24088873 – 24217983). Gene locus of *lncFKBPL* is shown in red. Bottom, changes of expression levels of genes shown in top panel upon *lncFKBPL* overexpression analyzed by qPCR. The error bars represent SEM. Student’s *t*-tests were used for statistical analysis.

A

lncFKBPL_WT · SV40pro · Luciferase · pA · *nonsense* · i-Control · i-*lncFKBPL*

	(a)	(b)	(c)	(d)	(e)	(f)	(g)	(h)	(i)	(j)	(k)	(l)	(m)	(n)	(o)	(p)	(q)	(r)	(s)	(t)	(u)	(v)	(w)
(a)																							
(b)	7.0e-03																						
(c)	2.9e-03	7.1e-01																					
(d)	9.8e-01	8.4e-03	4.6e-03																				
(e)	5.0e-03	4.7e-01	9.5e-01	5.2e-03																			
(f)	1.9e-03	9.5e-01	3.9e-01	3.1e-03	5.6e-01																		
(g)	1.2e-02	7.0e-02	5.2e-02	6.8e-03	3.8e-02	4.4e-02																	
(h)	1.4e-02	7.6e-02	3.2e-02	1.4e-02	5.9e-02	3.9e-02	6.9e-01																
(i)	6.9e-03	1.0e-02	2.0e-03	1.2e-02	1.3e-02	1.8e-04	6.4e-01	8.5e-01															
(j)	1.6e-02	8.4e-02	3.9e-02	1.5e-02	6.4e-02	4.5e-02	7.3e-01	6.5e-01	8.3e-01														
(k)	2.3e-02	1.2e-02	8.8e-02	2.6e-02	2.2e-02	6.9e-02	3.5e-01	3.5e-01	1.1e-01	3.7e-01													
(l)	9.6e-03	4.0e-03	2.8e-02	1.2e-02	2.4e-02	1.4e-02	2.5e-01	2.2e-01	2.2e-02	2.5e-01	7.4e-01												
(m)	9.3e-03	6.5e-03	1.4e-02	1.3e-02	2.0e-02	5.5e-03	3.6e-01	2.9e-01	2.2e-02	3.3e-01	5.9e-01	2.7e-02											
(n)	1.4e-02	4.2e-02	1.6e-02	1.9e-02	7.7e-02	3.3e-02	3.3e-01	2.4e-01	3.8e-02	2.7e-01	8.9e-01	9.1e-01	3.3e-01										
(o)	4.3e-02	6.8e-03	2.7e-03	5.2e-01	4.9e-03	1.7e-03	1.4e-02	1.6e-02	6.3e-03	1.8e-02	2.3e-02	9.3e-03	8.9e-03	1.4e-02									
(p)	1.4e-01	6.9e-03	2.9e-03	9.4e-01	4.9e-03	1.8e-03	1.2e-02	1.4e-02	6.7e-03	1.7e-02	2.3e-02	9.4e-03	9.1e-03	1.4e-02	2.5e-02								
(q)	2.7e-03	8.3e-01	6.2e-01	3.0e-03	1.6e-01	8.6e-01	3.3e-02	5.1e-02	8.0e-03	5.6e-02	5.2e-02	2.9e-02	2.0e-02	8.9e-02	2.7e-03	2.7e-03							
(r)	5.3e-03	1.3e-02	2.2e-02	7.3e-03	1.5e-02	4.1e-03	2.1e-01	2.0e-01	1.1e-02	2.2e-01	9.4e-01	6.1e-01	1.4e-01	9.1e-01	5.0e-03	5.1e-03	1.0e-02						
(s)	3.9e-02	6.3e-03	2.8e-03	1.8e-01	4.4e-03	1.5e-03	1.6e-02	2.1e-02	6.0e-03	2.4e-02	2.3e-02	8.7e-03	8.3e-03	1.4e-02	5.0e-02	3.0e-02	2.2e-03	4.3e-03					
(t)	2.8e-02	8.3e-03	3.0e-03	1.7e-01	6.3e-03	2.3e-03	1.5e-02	1.4e-02	8.7e-03	1.7e-02	2.8e-02	1.2e-02	1.1e-02	1.6e-02	7.5e-02	3.0e-02	3.7e-03	6.8e-03	9.4e-01				
(u)	4.9e-03	8.3e-01	5.4e-01	5.4e-03	6.6e-01	7.7e-01	4.4e-02	2.3e-03	3.5e-02	4.7e-03	2.9e-01	2.2e-01	1.4e-01	2.0e-01	5.2e-03	5.0e-03	8.5e-01	1.7e-01	6.4e-03	4.4e-03			
(v)	2.9e-02	2.1e-02	1.3e-02	1.2e-02	1.3e-02	1.0e-02	6.4e-03	3.0e-02	7.4e-02	2.8e-02	7.0e-02	4.0e-02	4.5e-02	5.6e-02	3.7e-02	2.9e-02	9.4e-03	2.8e-02	5.3e-02	4.3e-02	8.4e-03		
(w)	6.0e-03	8.5e-01	1.0e+00	4.1e-03	9.5e-01	8.0e-01	3.3e-03	3.5e-02	4.2e-02	3.3e-02	1.7e-01	1.6e-01	1.1e-01	2.1e-01	6.5e-03	6.0e-03	7.0e-01	1.1e-01	6.8e-03	7.2e-03	6.4e-01	4.2e-03	
(x)	7.3e-03	2.6e-02	1.6e-01	8.4e-03	1.1e-02	1.1e-01	9.9e-02	1.2e-01	1.9e-02	1.3e-01	5.9e-02	4.8e-02	3.5e-02	2.1e-01	7.2e-03	7.2e-03	4.5e-02	3.8e-02	6.5e-03	9.2e-03	5.6e-01	2.4e-02	2.9e-01

B

The mass spectrum result of the ~40KD silver staining band in Figure 4C

Gene_ID	Protein_Entry	Score	Matches	Sequences	Coverage
MED8	A0A5G2QHV8	653	37(16)	15(10)	31%
POLR3D	A0A4X1VG16	482	21(8)	12(7)	18%
POLR3E	A0A5G2QDR1	358	16(7)	9(6)	7%
CDK8	A0A287ALK4	318	16(6)	10(4)	10%
HNRNPD	A0A4X1TAE1	301	20(8)	11(5)	21%
HSPBP1	A0A5G2QLG3	264	8(3)	5(2)	12%
CCNC	A0A4X1VII9	248	7(3)	4(1)	10%
SQSTM1	A0A287BLE1	138	3(1)	2(1)	5%
PSME3	A0A5G2RIL9	128	2(0)	1(0)	9%
ACTR1A	A0A4X1TUJ6	113	3(0)	2(0)	6%
QKI	A0A8D0JDS3	96	1(0)	1(0)	3%
PSMC5	A0A8D1L3A4	86	2(0)	1(0)	2%
CORO1C	A0A4X1SR13	44	1(0)	1(0)	2%
DCTN3	A0A4X1UU88	18	1(0)	1(0)	4%
DNAJB1	A0A4X1V5G4	12	2(0)	1(0)	4%
SMAD2	A0A287BE69	11	1(0)	1(0)	3%

C

The mass spectrum result of the ~35KD silver staining band in Figure 4C

Gene_ID	Protein_Entry	Score	Matches		Coverage
MED4	A0A286ZZ81	361	32(14)	12(9)	38%
HNRNPD	A0A4X1TAE1	252	28(10)	12(6)	28%
MED7	A0A286ZZ81_PIG	203	10(4)	3(2)	22%
RNPS1	A0A286ZLK7_PIG	201	15(5)	7(4)	20%
SRP68	A0A4X1UYD0_PIG	192	16(7)	9(5)	12%
ATG3	A0A5G2R6H1_PIG	187	12(4)	7(3)	18%
SDCBP	A0A5G2R6P0_PIG	152	14(6)	8(3)	23%
NMRAL1	A0A286ZPF2_PIG	136	6(3)	2(1)	14%
PDRG1	A0A8D0XGF6_PIG	96	1(0)	1(0)	9%
MED27	A0A2C9F368_PIG	95	1(0)	1(0)	5%
PSMC5	A0A8D1L3A4_PIG	94	5(2)	2(0)	6%
SEC13	A0A4X1VUI0_PIG	86	6(2)	2(1)	16%
MED19	A0A286ZY58_PIG	78	9(3)	5(2)	19%
HAUS2	A0A4X1V192_PIG	61	2(0)	1(0)	12%
GTF2B	A0A287AL22_PIG	59	1(0)	1(0)	4%
DNAJB1	A0A4X1V5G4_PIG	35	4(1)	3(1)	6%

D

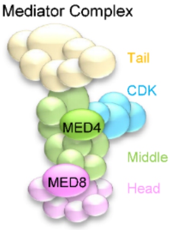

E

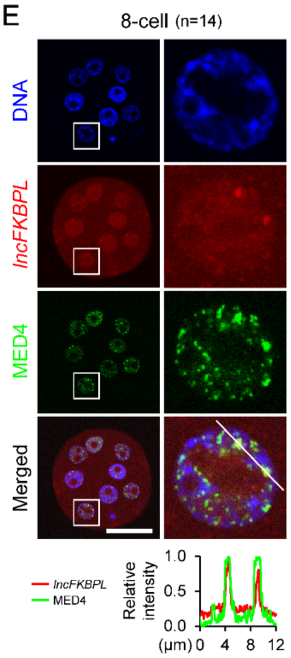

Supplementary Figure S4 *lncFKBPL* has an enhancer-like function.

A. The p values for Figure 4A. Student's t-tests were used for statistical analysis.

B, C. The mass spectrum results of the ~40 kDa (**B**) and the ~35 kDa (**C**) silver staining band in Figure 4C.

D. Illustration of the structure and components of Mediator complex. The Mediator complex has four regions, the head region, the middle region, the tail region, and the CDK region.

E. Top, lncFKBPL and MED4 are stained simultaneously by IF combined with RNA-FISH in pig eight-cell embryos. One nucleus marked by white square is magnified in the right lane. Bottom, line scans of the relative intensity of fluorescence signals indicated by the white dotted line in top panel.

A

The mass spectrum result of the ~90KD silver staining band in Figure 5A

Gene_ID	Protein_Entry	Score	Matches	Sequences	Coverage
HSP90AA1	A0A287AQK7_PIG	308	30(11)	24(9)	14%
PSMD14	A0A8D0LYU2_PIG	246	22(8)	14(5)	24%
HSP90AB1	A0A286ZKC5_PIG	235	23(8)	11(5)	12%
PPID	A0A4X1TWD1_PIG	199	11(4)	6(3)	19%
POU2F1	A0A8D0IZS7_PIG	184	7(2)	4(1)	6%
HSPA5	A0A287BIL8_PIG	179	9(3)	4(1)	9%
STUB1	A0A287BGG6_PIG	92	3(1)	3(1)	8%
KAT5	A0A287BIK4_PIG	89	2(1)	2(1)	4%
GSN	A0A287A6P1_PIG	79	6(3)	3(1)	4%
VRK3	A0A287AHL0_PIG	61	4(2)	3(1)	7%
EFTUD2	A0A8D1HBW5_PIG	43	1(0)	1(0)	1%
ATP5B	A0A480JUN1_PIG	32	1(0)	1(0)	2%
CXXC1	A0A4X1UJF4_PIG	22	2(1)	1(0)	2%
FBXL6	A0A287BC66_PIG	18	2(1)	1(0)	3%
ATP1A1	A0A4X1SUR0_PIG	13	1(0)	1(0)	1%

B

The mass spectrum result of the ~40KD silver staining band in Figure 5A

Gene_ID	Protein_Entry	Score	Matches	Sequences	Coverage
CDK9	A0A8D0S7Z0_PIG	232	22(10)	11(5)	24%
AIMP2	A0A8D1YYQ5_PIG	190	11(4)	8(3)	20%
EIF3H	A0A480JK41_PIG	162	14(5)	7(4)	24%
COPS5	A0A287AXW6_PIG	133	6(2)	3(1)	15%
BECN1	A0A4X1TY13_PIG	94	6(3)	3(2)	10%
HAX1	A0A8D0XZN0_PIG	72	6(2)	2(1)	9%
LONP1	A0A8D1N5F1_PIG	50	2(1)	1(0)	1%
TUFM	A0A8D1AZK6_PIG	49	4(1)	3(1)	8%
GSN	A0A287A6P1_PIG	41	3(1)	2(0)	3%
NR1H3	A0A8D1D3R7_PIG	29	3(1)	2(1)	4%
CDC37	A0A4X1V548_PIG	11	1(0)	1(0)	3%
RPLP0	A0A287AXW6_PIG	6	2(1)	1(0)	3%

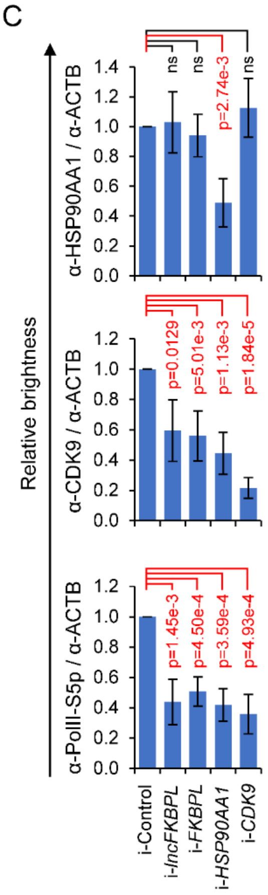

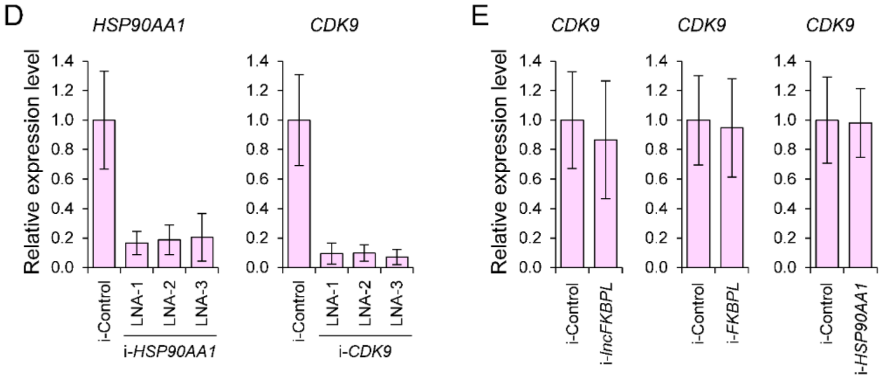

Supplementary Figure S5 FKBPL forms a complex with HSP90AA1 to stabilize CDK9 and then promote phosphorylation of RNA polymerase II.

A, B. The mass spectrum results of the ~90 KDa (**A**) and the ~40 KDa (**B**) silver staining band in Figure 5A.

C. Relative brightness of western blotting results in Figure 5D. ACTB works as internal reference. Three biological replicates were performed. The error bars represent SEM. Student's *t*-tests were used for statistical analysis.

D. RNA interference efficiency of LNA on *HSP90AA1*, *CDK9*. Three biological replicates were performed. The error bars represent SEM.

E. *CDK9* expression levels upon depletion of *lncFKBPL*, *FKBPL*, and *HSP90AA1*. Three biological replicates were performed. The error bars represent SEM.

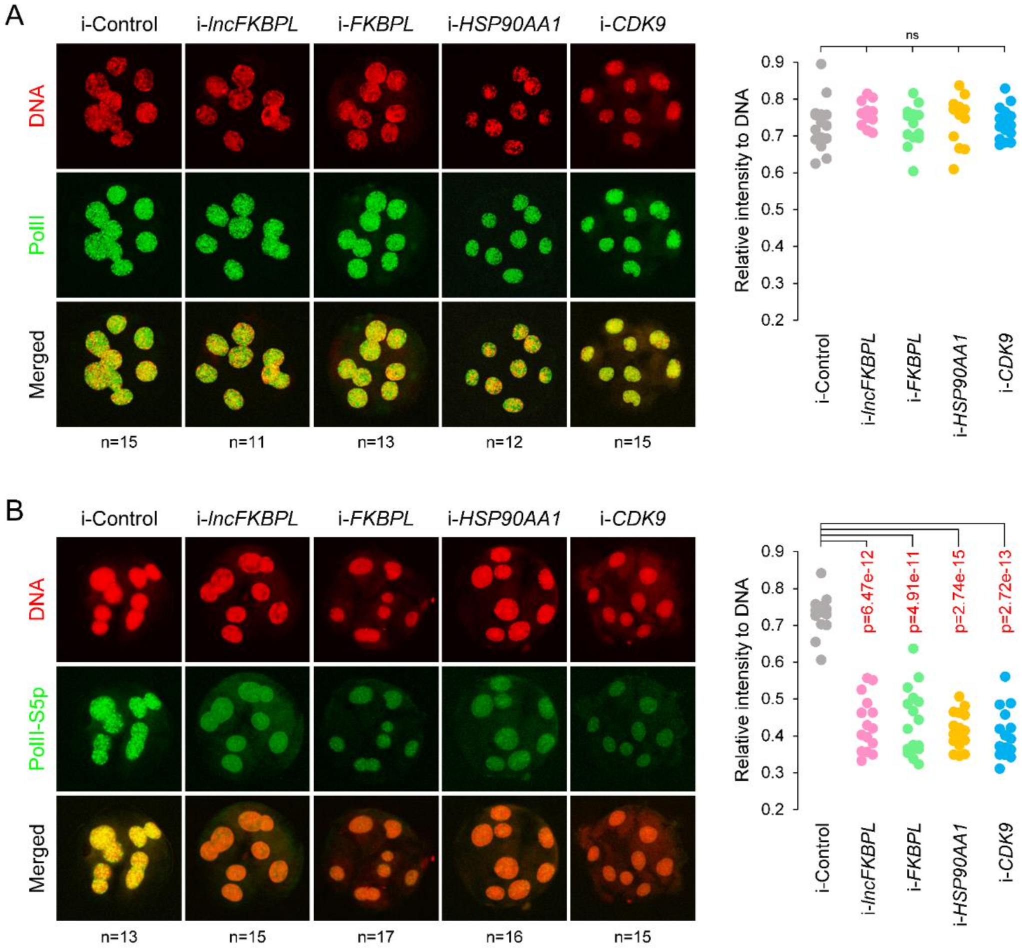

Supplementary Figure S6 Immunostaining results of polII and polII phosphorylation.

Left, Immunostaining results of polII (**A**) and polII phosphorylation (**B**) of pig eight-cell embryos upon *lncFKBPL*, *FKBPL*, *HSP90AA1*, or *CDK9* depletion. Right, relative intensity of polII (**A**) and polII phosphorylation (**B**) in the left panel.

Supplementary Tables

Supplementary Table 1 Oligos used in this paper.

Used for	Name	Sequence
Reverse transcription	RT-primer-R	AAGCAGTGGTATCAACGCAGAGTACTTTTTTTTTTTTTTTTTTTTTTTTTTTTVN
Semi-random PCR	Semi-random-F1	ACTGCTATCAC
	Semi-random-F2	TGGCTCTAGAT
	Semi-random-F3	AAATCATTAAC
	Semi-random-F4	CACGTAAAGCA
	Semi-random-F5	AATAGCTTCCA
	Semi-random-F6	GGGACCTTTAC
	Semi-random-F7	GGTGATGCCAA
	Semi-random-F8	AGAATGCATCC
	Semi-random-F9	GCCTTACAGAC
	Semi-random-F10	AGAACTGAGCT
	Semi-random-F11	TTTATAAGACC
	Semi-random-F12	AATGTGATGGT
	Semi-random-F13	TTGGCCTCCAG
	Semi-random-F14	CAGACACTGAA
	Semi-random-F15	TGGACTTGGCA
	Semi-random-F16	ACTGTGAGAAA
	Semi-random-F17	TGTTGTTTATA
	Semi-random-F18	GTATTCTGTTA
	Semi-random-F19	GGACTAAGACA
	Semi-random-F20	GTGATTAGGTC
	Semi-random-F21	ACTGAATCTGC
	Semi-random-F22	GCACCTTGATC
	Semi-random-F23	TGCTATAGACA
	Semi-random-F24	GATCGGAAGGA
	Semi-random-F25	GTTGAATATAC
	Semi-random-R	AAGCAGTGGTATCAACGCAGAGTAC
5'-RACE	lncFKBPL-5R-R1	TATTGACTGGGCTTGATAGCTCACC
	lncFKBPL-5R-R2	ACGTTTAGAGGACCAGCAGTATTGC
3'-RACE	PRRT1-3R-F1	CTAAACTAGGCCTCCACGGAAACTC
	PRRT1-3R-F2	AGCCTGGAGATGGGTCCCTAAAGC
RNA-FISH	lncFKBPL-probe-F	AGACTGAGTTGACTAAGACATTCTTCAACC
	lncFKBPL-probe-R	TAATACGACTCACTATAGGGAACTCCGCGATGGTAGTATTGACTGGGCTTG
qPCR	lncFKBPL-qF	ACTGATGAAATGACACTGAAAACATGC
	lncFKBPL-qR	TATTGACTGGGCTTGATAGCTCACC
	TNXB-qF	CGCACAGTGGCATCAACCTCAAGGTA
	TNXB-qR	GTCACTGGTCACAGGTGCTCACCTG
	ATF6B-qF	TCGGCCCAAGATGTCCCTGGTGATG
	ATF6B-qR	GTGGATCACCCTGGTGTCCATGACC
	FKBPL-qF	CAGAGCTGTGACCGGGTGCTGGA
	FKBPL-qR	CTGCCAGCACTTTCTTGAGGTCAGC
	PRRT1-qF	ACATGGTGTCAGCCGAGATCGCTTC
	PRRT1-qR	TACAGAGCACCATGGCTGCTATGCC

	PPT2-qF	TCCAATGGCTGGGATCTCCCACACA
	PPT2-qR	GAATCCGTCAGGAGAGCCAAGGTTCC
	EGFL8-qF	CCGGATCGAGTCTCTCAGTGACCAG
	EGFL8-qR	TGCAGAAGCACCTTATCTCCGCCGA
	AGPAT1-qF	TCGCAGTGCAGGCCCAGGTTCCCA
	AGPAT1-qR	CGGTGAGCATCGAGTGCCGGACTC
	RNF5-qF	GATTCCAGCCATTTGGCGACACTGG
	RNF5-qR	CCGACGAAATGGCTCGTGGGTATTG
	RNU6-qF	GTGCTCGCTTCGGCAGCACATATAC
	RNU6-qR	ATGGAACGCTTCACGAATTTGCGTG
	GAPDH-qF	GCTGGCATTGCCCTCAACGACCAC
	GAPDH-qR	TACTCCTTGGAGGCCATGTGGACCA
	TERRA-qF	CGGTTTGTTTGGGTTTGGGTTTGGGTTTGGGTTTGGGTT
	TERRA-qR	GGCTTGCCTTACCCTTACCCTTACCCTTACCCTTACCCT
Locked nucleotide acids (LNA)	Control-LNA	c*a*a*C*C*A*C*T*A*C*C*T*g*a*g*c
	lncFKBPL-LNA-1	t*g*a*C*T*G*G*G*C*T*T*G*a*t*a*g
	lncFKBPL-LNA-2	g*t*c*C*A*A*G*A*T*C*A*A*g*g*t*g
	lncFKBPL-LNA-3	t*t*t*A*G*G*G*G*T*A*A*G*a*t*t*c
	lncFKBPL1-LNA-1	g*t*t*T*A*T*A*A*C*C*T*A*g*a*a*a
	lncFKBPL1-LNA-2	a*g*c*A*T*T*T*A*C*A*A*A*c*a*g*a
	lncFKBPL1-LNA-3	c*a*g*A*C*A*G*C*T*T*G*G*a*t*t*a
	lncFKBPL2-LNA-1	t*a*c*T*C*T*A*C*T*G*C*A*a*c*g*a
	lncFKBPL2-LNA-2	a*c*c*T*T*T*G*G*A*A*A*T*g*c*t*c
	lncFKBPL2-LNA-3	a*t*t*C*T*T*T*C*A*A*T*A*a*t*g*t
	FKBPL-LNA1	t*a*a*T*T*C*G*A*A*C*T*A*t*a*a*g
	FKBPL-LNA2	a*t*t*T*T*C*T*A*G*A*A*T*t*t*g*g
	FKBPL-LNA3	a*a*t*T*A*G*C*C*A*A*A*C*a*t*c*t
	HSP90AA1-LNA-1	c*a*t*C*T*T*C*C*G*G*G*A*g*c*t*c
	HSP90AA1-LNA-2	a*c*t*T*T*G*T*C*T*T*C*T*t*c*t*c
	HSP90AA1-LNA-3	a*c*t*T*G*A*A*A*A*T*A*T*a*t*c*g
	CDK9-LNA-1	g*g*t*A*C*T*C*G*A*A*C*A*t*g*g*a
	CDK9-LNA-2	a*c*t*T*G*T*C*C*A*C*G*T*t*t*g*g
	CDK9-LNA-3	c*t*a*A*G*A*C*G*T*T*G*C*t*c*a*g
CRISPR-ON	T7-lncFKBPL1-sg-F	TAATACGACTCACTATAGGGTGCGGGTCTCTCTGGTATCCGT TTAAGAGCTATGCTGCGAATACG
	T7-lncFKBPL2-sg-F	TAATACGACTCACTATAGGGCAACAATAGACCCTATTTAAGT TTAAGAGCTATGCTGCGAATACG
	sgRNA-R	AAAAGCACCGACTCGGTGCCACTTTTTCAAGTTG
	T7-dCas9-F	TAATACGACTCACTATAGGGTGTCGTGACGTACGGCCACCAT GAGCCCCAAG
	VP64-R	CTCCACTGCCGCTAGCTAGTTAATCAGCATGTCCAGGTCGAA ATCATCAAG
	T7-MS2P-F	TAATACGACTCACTATAGGGTGTCGTGACGTACGGCCACCAT GGCTTCAAAC
	HSF1-R	TCTCCACTGCCGCTAGCTAGGAGACAGTGGGGTCCTTGGCTT TGGGAGGCTC

Note: for LNAs, the lower case letters represent locked nucletide acids, the upper case letters represent deoxynucleotides, and * represents phosphorothioate backbone.

Supplementary Table 2 Information from UCSC of 14 novel transcripts identified by random amplification.

Name	Chromosome	Strand	Genome locus	Upstream gene	Downstream gene	Length of RT-PCR results	Detected clones by sanger sequencing (%)
lncFKBPL	chr7	—	24180637-24181849	PRRT1	FKBPL	1213	3 (0.15)
aTPST2	chr14	+	44188734-44188806;<br>44190009-44190562	LOC106505959	CRYBB1	627	2 (0.1)
TUG1	chr14	+	47809416-47809900	MORC2	SMTN	485	2 (0.1)
RMST	chr10	—	86140073-86140386;<br>86144043-86144288;<br>86144387-86144549;<br>86152812-86153019;<br>86173516-86173720;<br>86207346-86207474;<br>86208517-86208617	NEDD1	LOC102160458	1364	2 (0.1)
aMTARC2	chr10	+	10145406-10146326;<br>10147762-10148089;<br>10150813-10150907;<br>10153386-10153615	LOC106505085	MTARC2	1574	2 (0.1)
MIAT	chr14	+	44252402-44254151	CRYBA4	MN1	1750	1 (0.05)
GAS5	chr9	—	116145161-116145243;<br>116145424-116145468;<br>116145656-116145685;<br>116146368-116146413	ZBTB37	DARS2	204	1 (0.05)
GAS5	chr9	—	116145169-116145468;<br>116146363-116146417;<br>116146611-116146640;<br>116146795-116146828;<br>116147095-116147134;<br>116147458-116147476;<br>116147619-116147634;<br>116147635-116147684	ZBTB37	DARS2	544	1 (0.05)
PRWN	chr2	+	51011969-51012413	PRSS38	WNT9A	445	1 (0.05)
SNPR	chr2	+	50928935-50929801	SNAP47	PRSS38	867	1 (0.05)
LIHS	chr6	—	58963693-58964937	LOC100624191	LOC100516019	1245	1 (0.05)
PDZD2-in	chr16	+	18039134-18039458	PDZD2 exon 1	PDZD2 exon 2	325	1 (0.05)
GGA3-in	chr12	—	6057493-6057802	GGA3 exon 2	GGA3 exon 1	310	1 (0.05)
DPYD-in	chr4	+	120202417-120202767	DPYD exon 2	DPYD exon 3	351	1 (0.05)
